# “On demand” redox buffering by H_2_S contributes to antibiotic resistance revealed by a bacteria-specific H_2_S donor†
†Electronic supplementary information (ESI) available. See DOI: 10.1039/c7sc00873b



**DOI:** 10.1039/c7sc00873b

**Published:** 2017-04-27

**Authors:** Prashant Shukla, Vinayak S. Khodade, Mallojjala SharathChandra, Preeti Chauhan, Saurabh Mishra, Shivakumara Siddaramappa, Bulagonda Eswarappa Pradeep, Amit Singh, Harinath Chakrapani

**Affiliations:** a Department of Microbiology and Cell Biology , Centre for Infectious Disease and Research , Indian Institute of Science , Bangalore 5600012 , Karnataka , India . Email: asingh@mcbl.iisc.ernet.in; b Department of Chemistry , Indian Institute of Science Education and Research Pune , Dr Homi Bhabha Road, Pashan , Pune 411 008 , Maharashtra , India . Email: harinath@iiserpune.ac.in; c Institute of Bioinformatics and Applied Biotechnology , Bengaluru 5600100 , Karnataka , India; d Sri Sathya Sai Institute of Higher Learning , Vidyagiri , Prasanthi Nilayam , Andhra Pradesh , India; e International Centre for Genetic Engineering and Biotechnology , New Delhi , India

## Abstract

Understanding the mechanisms of antimicrobial resistance (AMR) will help launch a counter-offensive against human pathogens that threaten our ability to effectively treat common infections. Herein, we report bis(4-nitrobenzyl)sulfanes, which are activated by a bacterial enzyme to produce hydrogen sulfide (H_2_S) gas. We found that H_2_S helps maintain redox homeostasis and protects bacteria against antibiotic-triggered oxidative stress “on demand”, through activation of alternate respiratory oxidases and cellular antioxidants. We discovered, a hitherto unknown role for this gas, that chemical inhibition of H_2_S biosynthesis reversed antibiotic resistance in multidrug-resistant (MDR) uropathogenic *Escherichia coli* strains of clinical origin, whereas exposure to the H_2_S donor restored drug tolerance. Together, our study provides a greater insight into the dynamic defence mechanisms of this gas, modes of antibiotic action as well as resistance while progressing towards new pharmacological targets to address AMR.

## Abstract 2

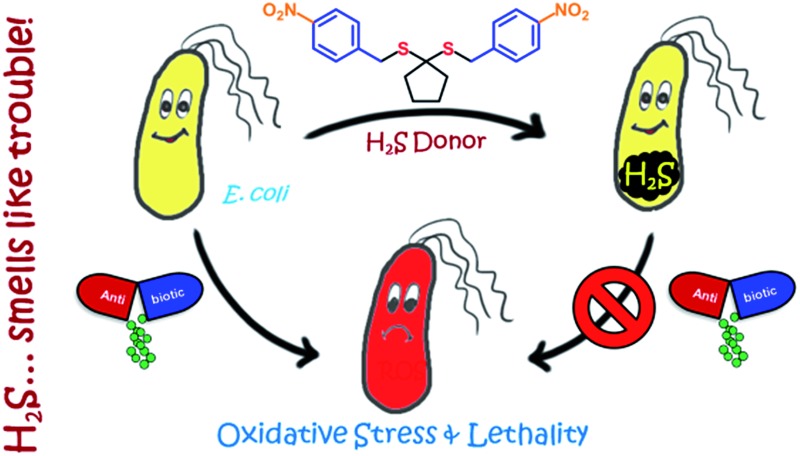
Enhancement of hydrogen sulfide in bacteria reveals a key role for this gas in mediating antibiotic resistance.

## Introduction

Maintenance of redox homeostasis is fundamental to cellular growth and survival. Induction of dysfunctional redox environment is a common mechanism used against pathogens by immune cells.^1^ In the past several decades, it has been well established that gases such as hydrogen sulfide (H_2_S) and nitric oxide (NO) affect cellular redox balance.^2^ Bacteria-derived-H_2_S through microbiota contribute significantly to repair mechanisms and are vital for the health of the gastrointestinal tract.^3^ Bacterial H_2_S is also implicated as a cytoprotective agent against antibiotic-induced stress, thereby enhancing antibiotic tolerance.^4^ Oxidant remediation by bacterial H_2_S is operational, but precise mechanisms of protection remain to be completely elucidated.^5^ Mapping out these cytoprotective mechanisms will help progress towards new strategies to combat the growing threat of antimicrobial resistance (AMR).^6^ Due to the dwindling arsenal of antibiotics, AMR is possibly the biggest problem that this current generation will face. In order to address this complex socioeconomic public health problem, multiple methodologies are necessary including a better understanding of the mechanisms of antibiotic action and factors contributing to antibiotic resistance.^7^ Herein, we systematically investigated the dynamic effects of H_2_S in protecting bacteria from antibiotic-induced stress and the role of H_2_S in modulating AMR.

**Fig. 1:**
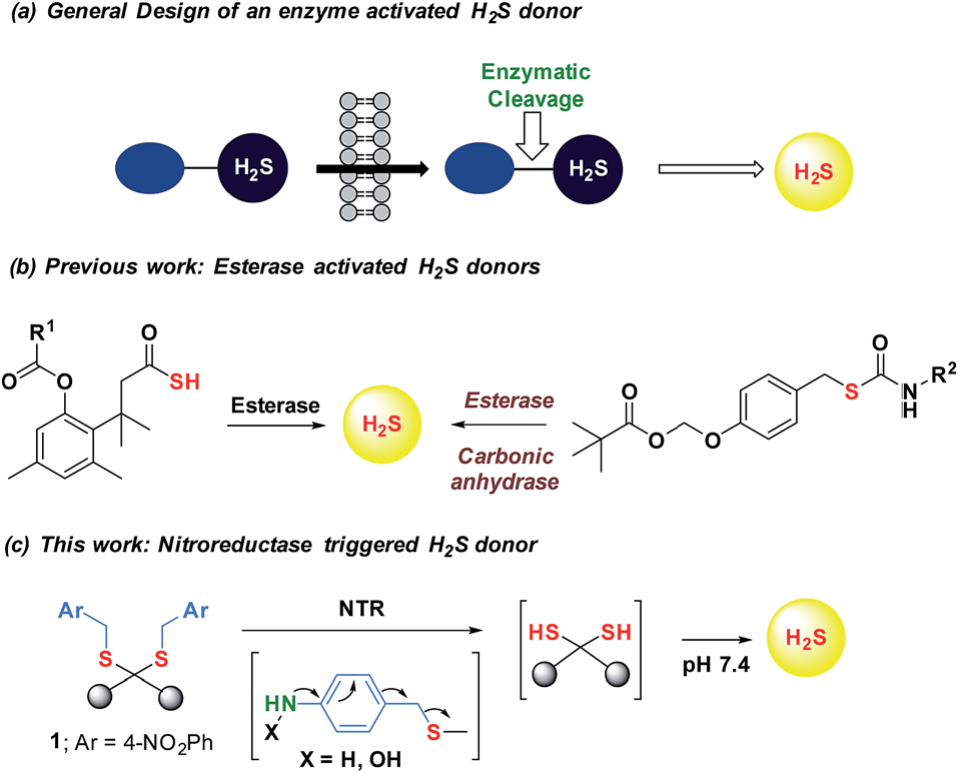
(a) General design of an enzyme activated H_2_S donor. (b) Esterase sensitive H_2_S donor; (b) esterase activated H_2_S and COS/H_2_S donor; R^1^ can be a non-steroidal anti-inflammatory drug (NSAID) while R^2^ is an aryl group or benzyl (c) bacterial enzyme nitroreductase (NTR) activated H_2_S donor and inset contains compounds synthesized in this study.

Being a gaseous species, reliable detection^8^ as well as controlled and site-specific generation of H_2_S within cells is fundamental to understanding its biology.^9^ Numerous donors of H_2_S (ref. 3) are in development but none, to our knowledge, distinguish one type of cells over others.^10^ Enzymes, as metabolic triggers for activation of donors, offer distinct advantages as they facilitate localization of H_2_S. A H_2_S generating functional group is tethered to a substrate for an enzyme that is normally expressed in cells of interest (Fig. 1a). Upon entry into cells, metabolism by the target enzyme frees up the active H_2_S generator inside cells thus achieving localized delivery. Recently, two esterase-activated H_2_S donors were reported with wide potential applications in cellular studies (Fig. 1b).^11^ However, generating H_2_S specifically in certain cells over others might be problematic when using esterase as a trigger. We chose *E. coli* nitroreductase (NTR), an oxygen-insensitive bacterial enzyme that is frequently expressed in bacteria but not in mammalian cells.^12^ Geminal dithiols are reported to undergo hydrolysis in buffer to produce H_2_S;^13^ 4-nitroaryl groups are known substrates for NTR. We hence designed **1**, an NTR-activated H_2_S donor (Fig. 1b).

## Results and discussion

**Fig. 2:**
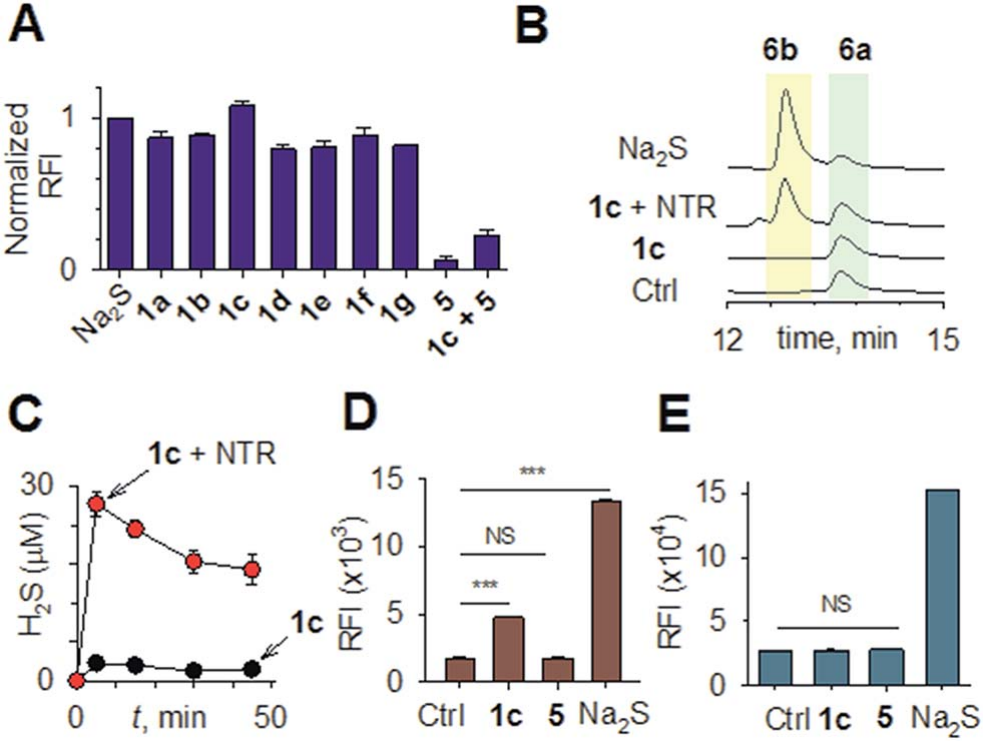
(a) Hydrogen sulfide generated during incubation of **1a–1g** (50 μM) with NTR in HEPES buffer pH 7.4 was estimated using a BODIPY-based sensor. (b) A monobromobimane (**6a**) method for the estimation of hydrogen sulfide. The formation of thioether **6b** supports the intermediacy of H_2_S. (c) Time course of H_2_S generation during incubation of **1c** in pH 7.4 buffer alone and in the presence of NTR was assessed by BODIPY-based sensor **4a**. (d) Flow cytometry analysis of intrabacterial H_2_S generation in *E. coli* using the probe **4a**. **1c** was incubated for 20 minutes ***p-value = 0.0001; NS = not significant. (e) Flow cytometry analysis of intracellular H_2_S generation in THP-1 cells. **1c** was incubated for 20 minutes (100 μM) and **4a** was used for H_2_S detection NS = not significant.

Synthesis of **1** is achieved by the reaction of a variety of ketones (**2**) with 4-nitrobenzyl thiol (**3**) (Table 1). A H_2_S-sensitive dye BODIPY-azide **4a** was employed to detect H_2_S. BODIPY-azide is known to be reduced by H_2_S to produce a fluorescent amine **4b**.^14^ Compounds **1a–1g** were independently exposed to NTR and all compounds were found to generate H_2_S under these conditions (Fig. 2a). The cyclopentyl derivative **1c** was found to be slightly better than the cohort of donors tested, and this compound was used for further studies.

**Table 1:** Synthesis of **1a–1g**

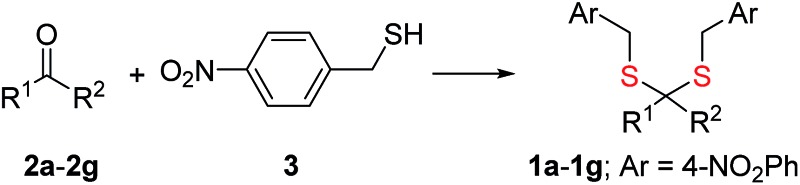					
Entry	R^1^	R^2^	Ketone	Prod	Yield %
1	CH_3_	CH_3_	**2a**	**1a**	80
2	Et	Et	**2b**	**1b**	78
3	Cyclopentyl		**2c**	**1c**	70
4	Cyclohexyl		**2d**	**1d**	71
5	Ph	CH_3_	**2e**	**1e**	60
6	4-FPh	CH_3_	**2f**	**1f**	42
7	Thiophene	CH_3_	**2g**	**1g**	38

A monobromobimane (mBBr, **6a**) assay (with some modifications) was next used to confirm the production of H_2_S.^15^ The electrophile mBBr reacts with sulfide anion to produce a thioether, which contains two bimane units (**6b**). When **6a** was treated with Na_2_S in a pH 7.4 buffer, as expected, **6b** was formed (Fig. 2b). Under similar conditions, when **1c** was co-incubated with **6a** and NTR, we found evidence for the formation of **6b** again supporting **1c** as a source of H_2_S when incubated with NTR (Fig. 2b). Next, NBD-fluorescein, a H_2_S sensitive dye, was synthesized and incubated in the presence of **1c** and NTR.^16^ Again, we found a distinct increase in fluorescence attributable to H_2_S generation (Fig. S1, ESI†). Thus, the formation of H_2_S was validated by several independent assays suggesting that this compound is a reliable donor of H_2_S. 
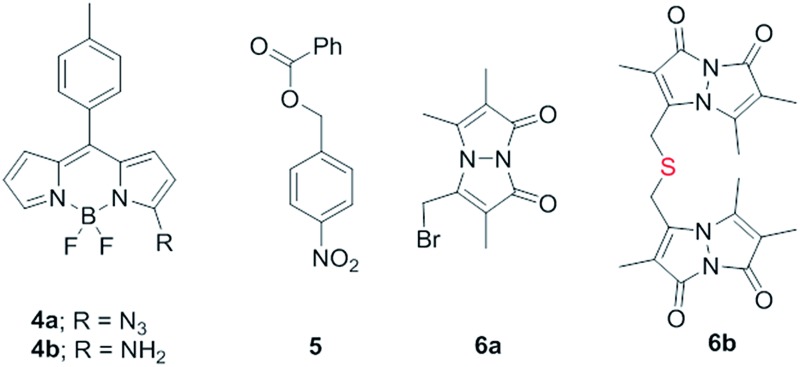


The H_2_S donor **1c** was able to maintain elevated levels of H_2_S over 45 min (Fig. 2c). The formation of a hydroxylamino- or amino-aryl derivative (Fig. 1c, Scheme S2, ESI†), which self-immolates to generate a geminal dithiol, was likely. This geminal dithiol should hydrolyze to produce H_2_S and a ketone.^13^ Accordingly, when **1e** was incubated in buffer in the presence of Zn and ammonium formate, acetophenone was formed, supporting the proposed mechanism (Fig. S2, ESI†).

Having established that **1c** generated H_2_S in cell-free conditions in the presence of a bacterial enzyme, the ability of this compound to permeate cells to be metabolized by NTR to generate H_2_S was evaluated. First, an HPLC-based method was used: *E. coli* cells were incubated with the H_2_S-sensitive dye **4a** and **1c**. Cells were lysed and HPLC analysis of the lysate revealed the formation of **4b** supporting H_2_S generation (Fig. S3, ESI†); a similar result was recorded for a variety of bacteria supporting the broad utility of this donor. Next, flow cytometry analysis revealed the generation of H_2_S inside intact bacterial cells when treated with **1c**, supporting the ability of this donor to enhance H_2_S levels in live bacterial cells (Fig. 2d). Next, 4-nitrobenzylbenzoate **5** (a likely competitive inhibitor) was synthesized using a previously reported method.^17^ This compound was by itself incapable of generating H_2_S in the presence of NTR and also inhibited H_2_S generation from **1c** (Fig. 2a). The negative control **5** did not generate H_2_S within the bacteria, suggesting that the metabolism of the nitro group does not contribute to H_2_S production (Fig. 2d). The H_2_S donor **1c** was ineffective in generating H_2_S in *E. coli* strains lacking NTR (Fig. S4, ESI†), confirming NTR-specificity *in vivo*. As NTR is predominantly produced in bacteria but not in mammalian cells, the H_2_S donor **1c** must selectively enhance H_2_S in bacteria. Human monocytic cells (THP-1) were treated with **1c**, and H_2_S levels were assessed by flow cytometry. Herein, we find that while Na_2_S was capable of enhancing H_2_S levels within THP-1 cells, **1c** remained completely ineffective (Fig. 2e). Thus, **1c** was selective in its ability to enhance H_2_S in bacteria over mammalian cells (Fig. S5, ESI†). To our knowledge, this is the first example of a H_2_S donor with species selectivity. Thus, this study lays the foundation for novel methodologies for site-specific enhancement of H_2_S using this class of H_2_S donors, for example, selectively enhancing H_2_S in microbiota to study the effects of this gas on colorectal cancer and other similar pathophysiologies is possible.^2^

**Fig. 3:**
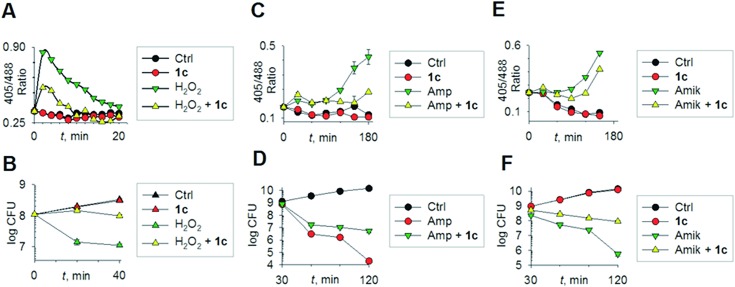
(a) Reduction–oxidation sensitive GFP (roGFP2) was used to measure dynamic changes in cytoplasmic redox potential of *E. coli* upon exposure to: H_2_O_2_, 1 mM; **1c**, 100 μM; (b) time-kill analysis of *E. coli* treated with hydrogen peroxide (1 mM) and **1c** (100 μM) during 40 min. (c) Dynamic changes in cytoplasmic redox potential of *E. coli* upon exposure to: Amp, 5 μg mL^–1^; **1c**, 100 μM; (d) time-kill analysis of *E. coli* treated with Amp (5 μg mL^–1^) and **1c** (100 μM) during 120 min. (e) Dynamic changes in cytoplasmic redox potential of *E. coli* upon exposure to: Amik, 20 μg mL^–1^; **1c**, 100 μM; (f) time-kill analysis of *E. coli* treated with Amik (20 μg mL^–1^) and **1c** (100 μM) during 120 min.

To begin understanding the mechanisms of H_2_S-mediated oxidation remediation, we used a non-invasive redox biosensor (roGFP2) and assessed dynamic changes in the cytoplasmic redox potential of *E. coli* in response to oxidative stress.^18^ An increase in 405/488 nm excitation ratio of roGFP2 indicates oxidative stress, while the reverse suggests reductive changes.^18*b*^ We first exposed *E. coli* expressing roGFP2 to **1c** and measured the roGFP2 biosensor response, and no significant changes in 405/488 ratio were observed (Fig. 3a). Hence, H_2_S alone did not affect ambient redox-potential of *E. coli*. In contrast, oxidative challenge with H_2_O_2_, a reactive oxygen species (ROS), rapidly increased the 405/488 ratio, and pre-treatment with **1c** significantly reversed this response (Fig. 3a). However, pre-treatment with either Na_2_S or **5** had no influence on H_2_O_2_-induced oxidative changes in the biosensor response (Fig. S6, ESI†).

Importantly, protective influence of **1c** on intrabacterial redox potential translated into significantly higher resistance displayed by *E. coli* against bactericidal concentrations of H_2_O_2_ (Fig. 3b). Interestingly, H_2_S itself did not have any significant effect on the growth of *E. coli*. Thus, intervention by H_2_S occurs when other endogenous oxidant–remediation systems are overwhelmed. This property is consistent with the lower reduction potential of H_2_S, when compared with major cellular thiols such as glutathione,^19^ and thus affords H_2_S a unique role in cellular redox chemistry. Furthermore, in contrast with other routinely used antioxidants in redox biology, such as thiourea and bipyridyl, **1c** does not significantly affect bacterial growth (Fig. 3b), suggesting that this tool would be appropriate for studying H_2_S-mediated response to dynamic redox alterations during antibiotic-induced stress and lethality.

The emerging model for antibiotic lethality involves the induction of complex redox and metabolic alterations as a consequence of drugs' interaction with their specific targets.^5,20^ Thus, it is important to understand the dynamic effects of H_2_S in mitigating antibiotic-induced redox stress.^21^ To accomplish this, we exposed *E. coli* to clinically relevant concentrations of the bactericidal antibiotic ampicillin (Amp; cell wall targeting), and an oxidative shift was recorded (Fig. 3c).^21a,22^ More importantly, pre-treatment with **1c** reduced the degree of oxidative shift induced by Amp, resulting in significant tolerance to antibiotics (Fig. 3d). Amp-mediated increase in roGFP2 ratios emerged earlier than the time points at which significant killing was observed, indicating that oxidative stress precedes death, and **1c**-derived H_2_S protects bacteria by maintaining cytoplasmic redox potential.

Similar results were recorded for amikacin (Fig. 3e–f), a translation inhibitor, and ciprofloxacin, a replication inhibitor (Fig. S7, ESI†). Altogether, these results demonstrate that elevating endogenous H_2_S levels can arrest antibiotics-triggered redox stress and killing.

**Fig. 4:**
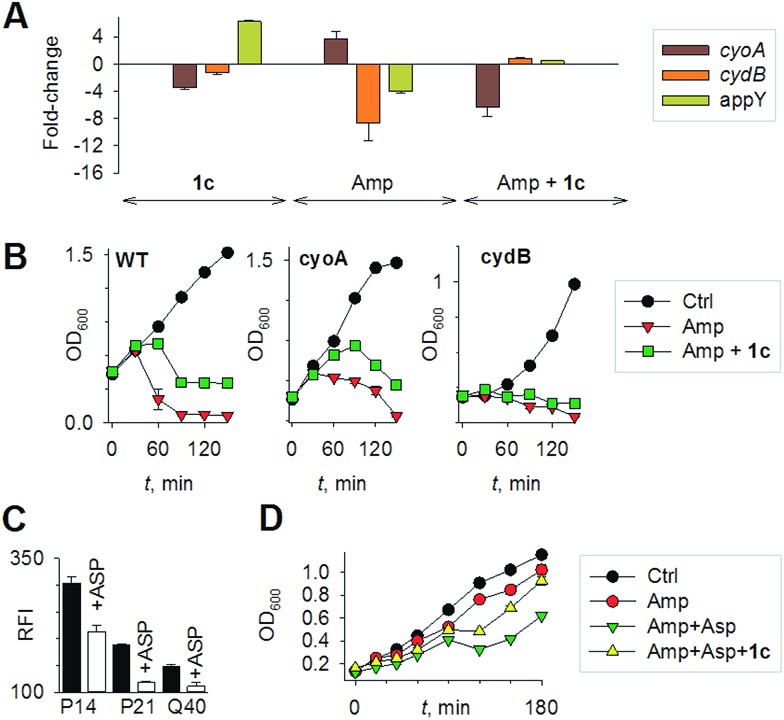
(a) qRT-PCR analysis of *E. coli* cells treated with **1c** alone, Amp alone. The fold-change is with respect to untreated control; **1c** + Amp where fold-change is with respect to Amp-treated cells. Fold change for each transcript was calculated by normalizing expression with the housekeeping 16SrRNA transcript. A 2-fold (*p* < 0.05) increase or decrease in expression was considered significant in all cases. (b) Growth curves of *E. coli* treated with Amp (5 μg mL^–1^) with and without **1c** (100 μM); Ctrl is untreated bacteria: wild-type (WT); cyoA mutant; cydB mutant. (c) H_2_S levels measured using the H_2_S-sensitive dye **4a** in the presence of ASP, a biosynthesis inhibitor of H_2_S generation (d) bacterial growth measurements: Amp (100 μg mL^–1^); Asp (4 mM) and **1c** (100 μM) were used in this study.

Mechanisms of H_2_S-mediated protection from antibiotic-induced lethality are poorly understood. It has been shown that H_2_S elevates cellular antioxidant capacity and suppresses iron load in order to mitigate antibiotic-linked ROS production.^23^ Since sulfide is a potent ligand of copper and heme moieties, H_2_S efficiently inhibits aerobic respiration by targeting copper-heme containing cytochrome bo oxidase (CyoA).^24^ Under these conditions, respiration becomes primarily dependent upon less energy-efficient cytochrome bd oxidase (CydB).^24^ Interestingly, modulation of cytochrome oxidases expression is known to influence antibiotic toxicity.^21*b*^ Therefore, we assessed whether terminal oxidases are important contributory factors in H_2_S-mediated antibiotic tolerance. First, quantitative reverse transcription-PCR (qRT-PCR) analysis of *E. coli* cells in the absence or presence of **1c** was conducted (see ESI†). A significant down-regulation of the genes encoding CyoA was observed with **1c**-treated bacteria (Fig. 4a). The expression of alternate oxidases was however either maintained (cytochrome bd oxidase I [*cydB*]) or highly induced (cytochrome bd oxidase II [*appY*]) by H_2_S (Fig. 4a). During growth under low-O_2_ tension, *E. coli* down-regulated *cyo* operon and upregulated *cyd* and *app* operons, indicating that H_2_S triggered genetic and physiological changes comparable to O_2_-limitation.^25^ Amp treatment reversed the influence of H_2_S on the expression of cytochrome oxidases as *cyoA* and *cydB* transcripts were significantly induced and repressed, respectively, compared to untreated cells (Fig. 4a). The *appY* transcript remained down-regulated in response to Amp. Data suggest that Amp treatment promotes respiration *via* energetically efficient CyoA, which is consistent with a recent study demonstrating acceleration in aerobic respiration in response to bactericidal antibiotics.^21*b*^

Having observed divergent effects of H_2_S and Amp on cytochrome oxidases gene expression, we next examined the outcome of H_2_S and Amp combination on transcription. qRT-PCR analysis of *E. coli* pre-treated with **1c**, followed by exposure to Amp showed severely down-regulated expression *cyoA*, whereas expression of *cydB* and *appY* was robustly maintained (Fig. 4a) compared to that of Amp alone. Thus, maintenance of a respiratory flux through cytochrome bd oxidase I/II in response to H_2_S treatment may be a key trait that permits adaptation upon subsequent exposure to antibiotics. To examine this possibility, we assessed cell-killing in respiratory mutants lacking either *cyoA* or *cydB*. While **1c**-pretreatment resulted in a significant attenuation of Amp lethality in the case of CyoA mutant (like WT strain), it was completely ineffective in protecting *cydB* mutant (Fig. 4b). In LB medium, WT, *cyoA*, and *cydB* strains showed comparable growth profiles in the absence or presence of **1c**, indicating that the differences in Amp susceptibility are not a consequence of reduced growth rates. Sustenance of *cydB* expression in response to H_2_S-Amp combination, coupled with maintenance of H_2_S-mediated antibiotic tolerance in *cyo* mutant (where aerobic respiration is mainly carried out by CydB) but not in *cydB* mutant, suggests that the H_2_S effect is likely to be dependent upon *cydB*.^24^ Along with its role in respiration, CydB from *E. coli* has been shown to reduce H_2_O_2_ by displaying catalase and quinol peroxidase activities.^26^ Therefore, maintenance of *cydB* expression by H_2_S can potentiate antibiotic tolerance by bolstering the bacterial antioxidant capacity. In concurrence with this result, displayed heightened sensitivity to H_2_O_2_ compared to *cyoA* and WT strains (Fig. S8, ESI†).

**Fig. 5:**
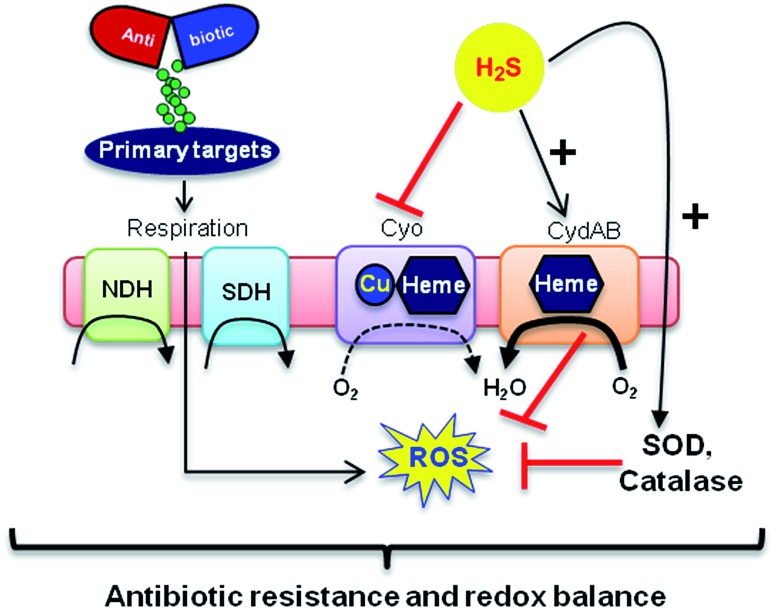
Evolving model for H_2_S mediated antibiotic tolerance. Bactericidal antibiotics alters respiration and metabolism to elevate the production of endogenous ROS. Based on our findings, we propose that bacterial H_2_S provides tolerance to antibiotics by two mechanisms; (i) down-regulation of energy-efficient cytochrome bo oxidase (Cyo) and induction of less-energy efficient cytochrome bd oxidase I/II (CydAB/AppBC) to maintain respiratory flux and redox balance, and (ii) augmentation of antioxidant capacity by elevating catalase and superoxide dismutase (SOD) activities. NDH: NADH dehydrogenase and SDH: succinate dehydrogenase.

In addition, consistent with previous results,^4*d*^ we observed that H_2_S is ineffective in protecting cells that lack cytoplasmic catalase and peroxidase (KatA, KatE, ahpCF; HPX-) from Amp-induced lethality (Fig. S9, ESI†). Altogether, data implicate a central role for cytochrome bd oxidase and oxidant-remediation mechanisms in diminishing the effectiveness of the antibiotic by H_2_S (Fig. 5).^27^

Finally, in order to examine if elevated endogenous H_2_S levels are associated with drug resistance in the physiological context of human infections, we measured the intracellular H_2_S levels of several multidrug-resistant (MDR) *E. coli* strains isolated from patients (Table S2, ESI† for resistance profile) suffering from urinary tract infections (UTI). The endogenous H_2_S levels were considerably higher than WT indicating a possible functional role for H_2_S in antibiotic resistance (Fig. S10, ESI†).^28^ In the presence of a well-established 3-mercaptopyruvates sulfurtransferase (3-MST) inhibitor (aspartate, Asp), we found that H_2_S levels significantly diminished (Fig. 4c).^4*d*^ To understand the functional relevance of endogenous H_2_S levels in drug resistance, we monitored resistance of P14 strain to Amp. We found that pre-treatment with Asp efficiently inhibited the growth in response to Amp (Fig. 4d). More-importantly, co-treatment with Asp and **1c**, significantly restored resistance to Amp in the strain P14 (Fig. 4d). Altogether, these findings revealed a previously unknown contribution of H_2_S in cooperating with the genetic mechanisms of antibiotic resistance (Fig. 5). Further study is needed to examine H_2_S-mediated mechanisms contributing to the emergence of drug-resistance in clinical strains.^5,28^ Amongst the major infectious diseases, UTI affects millions and is further complicated by conditions such as diabetes. *E. coli* has now become resistant to most major classes of antibiotics and therefore there is an urgent need to develop new therapeutics. Recently, Berkowitz and co-workers developed a CBS inhibitor that helps to prevent the deleterious effects of enhanced H_2_S such as neuronal cell death during episodes of stroke.^29^ The inhibitors that were developed in their study showed a marked diminution in neuronal cell death compared with an untreated control. It is likely that inhibitors of 3-MST may find similar application in sensitizing resistant pathogens.^30^ Our results provide a sound pharmacological basis for the design of inhibitors of biosynthesis of H_2_S as a possible adjuvant.^29–31^ Furthermore, we identified critical aspects of bacterial physiology that could be exploited as part of new potentiation strategies. For examples, targeting antioxidant enzymes and alternate respiratory complexes (Cyd/App) is likely to enhance the killing potential of antibiotics. A combination of molecules/drugs targeting H_2_S, antioxidants, and respiration could have a remarkable impact on drug-resistance and clinical outcomes.

## Conclusions

In summary, we report a new H_2_S donor that reliably and selectively enhances H_2_S within bacteria. An application of our new tool clearly revealed that H_2_S is a key player in the maintenance of intracellular redox balance of bacteria to counteract a lethal degree of oxidative stress induced by antibiotics. The critical role that H_2_S played in modulating drug resistance is also shown. Antibiotic resistance is emerging as possibly the biggest global health challenge for this generation. Therefore understanding pathogen defence mechanisms and their consequences in drug resistance is critical. From the evolutionary perspective, H_2_S generating enzymes are prevalent in most sequenced bacterial genomes including environmental bacteria, indicating a naturally conserved role of H_2_S in ensuring survival. It is likely that H_2_S producing capability is under the selective pressure in diverse environmental bacteria that arises due to antimicrobials secreted by other bacteria and fungi inhabiting the same niche. Our study presents significant advances towards a complete understanding of antibiotic-induced stress and cytoprotective mechanisms of H_2_S.
