# Supplementary material for: “On demand” redox buffering by H2S contributes to antibiotic resistance revealed by a bacteria-specific H2S donor
Source: Chem Sci. 2017 Apr 27;8(7):4967–72. doi: 10.1039/c7sc00873b (PMC5607856; doi:10.1039/c7sc00873b)

Electronic Supplementary Material (ESI) for Chemical Science.
This journal is © The Royal Society of Chemistry 2017

Electronic Supplementary Information (ESI)

for

"On Demand" Redox Buffering by H_2S Contributes to Antibiotic Resistance Revealed by a Bacteria-Specific H_2S Donor

Prashant Shukla,[a,e]† Vinayak S. Khodade,[b]† Mallojjala SharathChandra,[b] Preeti Chauhan,[b] Saurabh Mishra,[a] Shivakumara Siddaramappa,[c] Bulagonda Eswarappa Pradeep[d], Amit Singh[a,]* and Harinath Chakrapani[b,]*

a. Department of Microbiology and Cell Biology & Centre for Infectious Disease and Research, Indian Institute of Science, Bangalore 5600012, Karnataka, India. E-mail: asingh@mcbl.iisc.ernet.in
b. Department of Chemistry, Indian Institute of Science Education and Research Pune, Dr. Homi Bhabha Road, Pashan Pune 411 008, Maharashtra, India. E-mail: harinath@iiserpune.ac.in
c. Institute of Bioinformatics and Applied Biotechnology Bengaluru 5600100, Karnataka, India
d. Sri Sathya Sai Institute of Higher Learning, Vidyagiri, Prasanthi Nilayam, Andhra Pradesh, India
e. International Centre for Genetic Engineering and Biotechnology, New Delhi, India

† These authors contributed equally

Experimental Procedures

General Methods. All chemicals were purchased from commercial sources and used as received unless stated otherwise. Petroleum ether (PE) and ethyl acetate (EtOAc), for chromatography were distilled before use. THF and DCM were dried using a sodium wire and distilled before use. Column chromatography was performed using Merck silica gel (60-120/100–200 mesh) as the solid support. ^1H and ^{13}C spectra were recorded on a JEOL 400 MHz (or 100 MHz for ^{13}C) or BRUKER 500 MHz spectrometer using either the residual solvent signal as an internal reference ($CDCl_3$ δH, 7.26 ppm, δC 77.1 ppm) or a tetramethylsilane (δH = 0.00, δC = 0.0) standard. Chemical shifts (δ) are reported in ppm. The following abbreviations are used: s (singlet), br s (broad singlet), d (doublet), t (triplet) and q (quartet). Mass spectra were obtained using a HRMS-ESI-Q-Time of Flight LC-MS (Synapt G2, Waters) or MALDI TOF/TOF Analyser (Applied Biosystems 4800 Plus). Infrared spectra (IR) were obtained using NICOLET 6700 FT-IR spectrophotometer using a KBr disc. Melting points were measured using a VEEGO melting point apparatus in open glass capillary and values reported are uncorrected. High performance liquid chromatography (HPLC) was performed on Agilent Technologies 1260Infinity, attached with a C-18 column (Phenomenex, 5 μm, 4.6 × 250 mm). Fluorimetric and spectrophotometric measurements were performed using Thermo Scientific Varioscan microwell plate reader. The purity of all compounds synthesized in this study was ≥ 95% as determined by HPLC and/or elemental analysis. All cellular experiments were performed in compliance with the relevant laws and institutional guidelines, and the institutional committee has approved the experiments.

Synthesis and Characterization. Compounds **3**[1]**, 4a**[2] and **5**[3] have been previously reported and analytical data that we collected were consistent with the reported values.

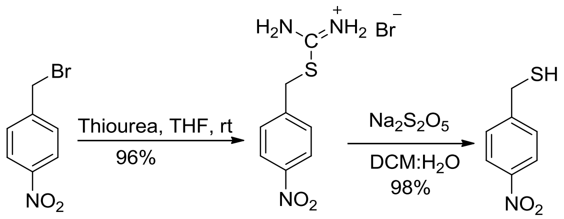

3

Scheme S1. Synthesis of **3**

General procedure for synthesis of 1a-1g. To a solution of ketone **2a-2g** (1 mmol) and *p*-nitrobenzylmercaptan **3** (2.5 mmol) in chloroform (5 mL) was added 0.5 equivalents of $TiCl_4$ at 0 °C. The resulting reaction mixture was allowed to warm up to rt and stirred overnight or until the consumption of the corresponding ketone (monitored by TLC). To the resulting heterogeneous mixture was then added brine (5 mL) and aqueous layer was extracted with $CHCl_3$ (3×10 mL). The combined organic layer was dried over anhydrous sodium sulfate, filtered and concentrated. The crude material was purified using silica gel column chromatography, eluted with 4-8% EtOAc/pet ether to afford **1a-1g**.

Propane-2,2-diylbis((4-nitrobenzyl)sulfane) 1a: Following the general procedure for the synthesis of bis(4-nitrobenzyl)sulfane **1a** (260 mg, 80%) was obtained as a colorless solid: mp 148-150 °C; FT-IR (ν_{max}, cm^{-1}): 3742, 1647, 1596, 1334, 1103, 853, 795, 713; ^{1}H NMR (400 MHz, $CDCl_3$): δ 8.17 (d, *J*= 8.7 Hz, 4H), 7.50 (d, *J*= 8.7 Hz, 4H), 3.92 (s, 4H), 1.60 (s, 6H); ^{13}C NMR (100 MHz, $CDCl_3$): δ 147.1, 145.9, 130.0, 123.9, 58.2, 34.8, 30.9; HRMS (ESI): calcd. For $C_{17}H_{18}N_2O_4S_2$ $[M+Na]^+$: 401.0605; Found: 401.0605

Pentane-3,3-diylbis((4-nitrobenzyl)sulfane) 1b: Following the general procedure for the synthesis of bis(4-nitrobenzyl)sulfane **1b** was obtained with an yield of 57% (370 mg). MP 124-126 °C; FT-IR (ν_{max}, cm^{-1}): 1658, 1598, 1559, 1517, 1458, 1342, 1106, 1013, 855; ^{1}H NMR (500 MHz, $CDCl_3$): δ 8.17 (d, *J* = 8.6 Hz, 4H), 7.48 (d, *J* = 8.7 Hz, 4H), 3.87 (s, 4H), 1.67 (q, *J* = 7.3 Hz, 4H), 0.93 (t, *J* = 7.3 Hz, 6H); ^{13}C NMR (50 MHz, $CDCl_3$): δ 147.0, 145.8, 129.9, 123.8, 68.5, 33.4, 28.6, 8.3; HRMS (ESI): calcd. For $C_{19}H_{22}N_2O_4S_2$ $[M+Na]^+$: 429.0919; Found: 429.0921

Cyclopentane-1,1-diylbis((4-nitrobenzyl)sulfane) 1c: Following the general procedure for the synthesis of bis(4-nitrobenzyl)sulfane **1c** was obtained with an yield of 70% (169 mg). MP 94-96 °C; FT-IR (ν_{max}, cm^{-1}): 3852, 3734, 3674, 3648, 1716, 1597, 1558, 1540, 1508, 1338, 1107, 853, 799; ^{1}H NMR (400 MHz, $CDCl_3$): δ 8.17 (d, *J*= 8.7 Hz, 4H), 7.50 (d, *J*= 8.6 Hz, 4H), 3.92 (s, 4H), 1.87-1.84 (m, 4H), 1.78-1.75 (m, 4H); ^{13}C NMR (100 MHz, $CDCl_3$): δ 147.1, 146.2, 130.1, 123.9, 67.9, 41.6, 35.3, 24.3; HRMS (ESI): calcd. For $C_{19}H_{20}N_2O_4S_2$ $[M+Na]^+$: 427.0761; Found: 427.0762

Cyclohexane-1,1-diylbis((4-nitrobenzyl)sulfane) 1d: Following the general procedure for the synthesis of bis(4-nitrobenzyl)sulfane **1d** was obtained with an yield of 71% (140 mg). MP 96-98 °C; FT-IR (ν_{max}, cm^{-1}): 3852, 3734, 3674, 3648, 1733, 1716, 1698, 1652, 1508, 1107, 854, 745; ^{1}H NMR (400 MHz, $CDCl_3$): δ 8.17 (d, *J* = 8.6 Hz, 4H), 7.50 (d, *J*= 8.6 Hz, 4H), 3.90 (s, 4H), 1.84-1.81 (m, 4H), 1.62-1.58 (m, 4H), 1.47-1.45 (m, 2H); ^{13}C NMR (100 MHz, $CDCl_3$): δ 147.1, 146.2, 130.0, 123.9, 64.4, 38.0, 33.3, 25.5, 22.5; HRMS (ESI): calcd. For $C_{20}H_{22}N_2O_4S_2$ $[M+Na]^+$: 441.0919; Found: 441.0923.

(1-Phenylethane-1,1-diyl)bis((4-nitrobenzyl)sulfane) 1e: Following the general procedure for the synthesis of bis(4-nitrobenzyl)sulfane **1e** was obtained with an yield of 60% (220 mg). MP 109-111 °C; FT-IR (ν_{max}, cm^{-1}): 3586, 1558, 1396, 974, 857, 803, 767, 752, 699; ^{1}H NMR (400 MHz, $CDCl_3$): δ 8.10 (d, *J*= 8.7 Hz, 4H), 7.69 (d, *J*= 7.6 Hz, 2H), 7.40-7.22 (m, 7H), 3.88-3.77 (m, 4H), 2.04 (s, 3H); ^{13}C NMR (100 MHz, $CDCl_3$): δ 147.0, 145.2, 142.2, 130.0, 128.7, 128.1, 127.1, 123.8, 62.7, 35.4, 30.5; HRMS (ESI): calcd. For $C_{22}H_{20}N_2O_4S_2$ $[M+Na]^+$: 463.0761; Found: 463.0768.

(1-(4-Fluorophenyl)ethane-1,1-diyl)bis((4-nitrobenzyl)sulfane) 1f: Following the general procedure for the synthesis of bis(4-nitrobenzyl)sulfane **1f** was obtained as a semisolid compound with an yield of 42% (274 mg); FT-IR (ν_{max}, cm^{-1}): 1598, 1518, 1345, 1163, 1065, 858, 841; ^{1}H NMR (500 MHz, $CDCl_3$): δ 8.12 (d, *J*= 8.7 Hz, 4H), 7.69-7.66 (m, 2H), 7.37 (d, *J* = 8.7 Hz, 4H), 7.02 (t, *J* = 8.6 Hz, 2H), 3.89-3.73 (m, 4H), 2.02 (s, 3H); ^{13}C NMR (50 MHz, $CDCl_3$): δ 159.7, 147.0, 144.9, 138.4, 129.9, 129.0, 128.8, 123.8, 115.6, 115.2, 62.0, 35.4, 30.5; HRMS (ESI): calcd. For $C_{22}H_{29}FN_2O_4S_2$ $[M+Na]^+$: 481.0667; Found: 481.0671.

2-(1,1-Bis((4-nitrobenzyl)thio)ethyl)thiophene 1g: Following the general procedure for the synthesis of bis(4-nitrobenzyl)sulfane **1g** was obtained as a semisolid compound with an yield of 38% (266 mg); FT-IR (ν_{max}, cm^{-1}): 1599, 1522, 1347, 1109, 1077, 857, 803, 739; ^{1}H NMR (500 MHz, $CDCl_3$): δ 8.13 (d, *J*= 8.7 Hz, 4H), 7.40 (d, *J*= 8.7 Hz, 4H), 7.32-7.28 (m, 1H), 7.16 (dd, *J* = 3.16, 1.1 Hz, 1H), 6.94-6.92 (m, 1H), 3.93-3.86 (m, 4H), 2.07 (s, 3H); ^{13}C NMR (50 MHz, $CDCl_3$): δ 148.4, 147.0, 144.9, 130.0, 126.8, 126.3, 126.1, 123.8, 59.5, 35.8, 31.9; HRMS (ESI): calcd. For $C_{20}H_{18}N_2O_4S_3$ $[M+Na]^+$: 469.0326; Found: 469.0327.

Calibration curve of Na_2S: A stock solution of sodium sulfide 10 mM was prepared in water. A standard solutions of Na_2S (20, 40, 60, 80, 100 μM) were prepared by diluting 20, 40, 60, 80 and 100 μL of 1mM Na_2S to 1 mL of water, respectively. 180 μL of standard solutions of Na_2S and 20 μL (1 mM) azo-BODIPY **4a** was added to 96 well plate in triplicate. The resulting mixture was incubated at 37 °C for 10 min and fluorescence (excitation 444 nm; emission 520 nm) from reaction mixture was measured using microtiter plate reader.

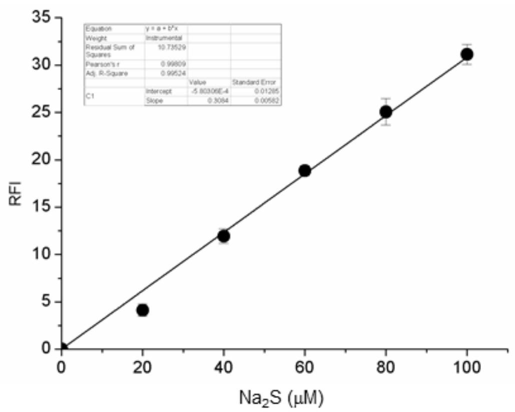

Measurement of H_2S using BODIPY-based dye 4a: A stock solution of **1a-1g** (2.5 mM) was prepared in DMSO and 10 mM NADH was prepared in HEPES buffer pH 7.4. A stock solution of commercially available *E. coli* nitroreductase (NTR) was prepared using 1 mg of a lyophilized powder dissolved in HEPES buffer (2 mL). A solution of **4a** (1 mM) was prepared in DMSO and stored under dark conditions. The reaction mixture was prepared by adding **1a-1g** (4 μL, 2.5 mM), NADH (10 μL, 10 mM) and NTR (4 μL stock as previously prepared) in HEPES buffer of pH 7.4 (162 μL). The resulting mixture was incubated at 37 °C for 5 min. 20 μL of **4a** (1 mM) was added to the above mixture and incubated for 10 min at 37 °C under dark conditions and fluorescence (excitation 444 nm; emission 520 nm) from reaction mixture was measured using microtiter plate reader. In the control experiment **1c** was co-treated with competitive substrate of nitroreductase **5** (5 μL, 20 mM). Data presented are an average of three independent experiments. The yields reported are determined by a dose-response curve generated with Na_2S (see above).

Detection of H_2S from 1c using mBBr assay: A 10 mM stock solution of mBBr was prepared in degassed CH_3CN and stored at -20 °C under dark conditions. A 100 mM sodium sulfide solution was prepared in degassed water and diluted it further to 1 mM in degassed water. Reaction mixture was prepared by adding sodium sulfide (30 μL, 1 mM), reaction buffer (70 μL, 100 mM Tris-HCl buffer pH 9.5 with 0.1 mM DTPA) and

mBBr (50 μL, 0.4 mM) under ambient conditions. The resulting mixture was incubated at room temperature for 30 min under dark conditions and quenched with 50 μL (1M HCl) solution. In the experiments of **1c** with NTR, the reaction mixture was prepared by adding **1c** (4 μL, 2.5 mM), NADH (10 μL, 10 mM) NTR (4 μL as prepared previously) in reaction buffer (82 μL) pH 9.5. The resulting mixture was incubated for 10 min at 37 °C. To this incubated mixture, mBBr (50 μL, 0.4 mM) was added and incubated at rt for 30 min under dark conditions. The mixture was quenched with 50 μL (1 M HCl) solutions. The resulting mixture was filtered (0.22 μm) and injected (10 μL) in an Agilent high performance liquid chromatography (HPLC) attached with a fluorescence detector (excitation at 390 nm; emission at 475 nm). The column used was Zorbax SB C-18 reversed phase column (250 mm × 4.6 mm, 5 μm). The mobile phase was water: acetonitrile containing 0.1 % trifluroacetic acid and a gradient starting with 85: 15 →0 min, 85: 15 to 65: 35 → 0 - 5 min, 65: 35 to 45: 55 → 5 - 16 min, 45: 55 to 30: 70 →16 – 23 min, 30: 70 to 10: 90 →23 – 24 min, 10: 90 to 85: 15 →24 – 26 min was used with flow of 0.6 mL/min. Under these conditions, we observed the formation of sulfide dibimane, which elutes at 12.78 min.

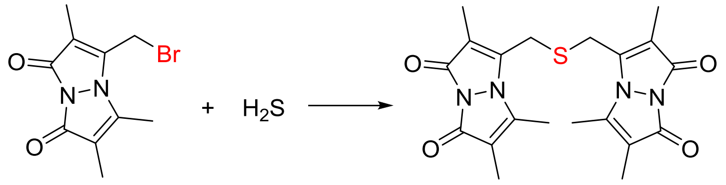

6a **6b**

Measurement of H_2S using NBD-fluorescein: Stock solutions of **1c** and **5** (2.5 mM) were prepared in DMSO and 10 mM NADH was prepared in HEPES buffer pH 7.4. A stock solution of commercially available *E. coli* nitroreductase (NTR) was prepared using 1 mg of a lyophilized powder dissolved in HEPES buffer (2 mL). A solution of NBD-fluorescein (1 mM) was prepared in DMSO and stored under dark conditions. The reaction mixture was prepared by adding **1c** or **5** (4 μL, 2.5 mM), NBD- fluorescein (2 μL, 1 mM), NADH (10 μL, 10 mM) and NTR (8 μL stock as previously prepared) in HEPES buffer of pH 7.4 (176 μL). The resulting mixture was incubated at 37 °C for 2 h. Fluorescence (excitation 490 nm; emission 514 nm) from reaction mixture was measured using microtiter plate reader. In the control experiment NTR + NADH were co-incubated with NBD-fluorescein dye and the fluorescence value was subtracted from each data point (except for Na_2S). Data presented are an average of three independent experiments.

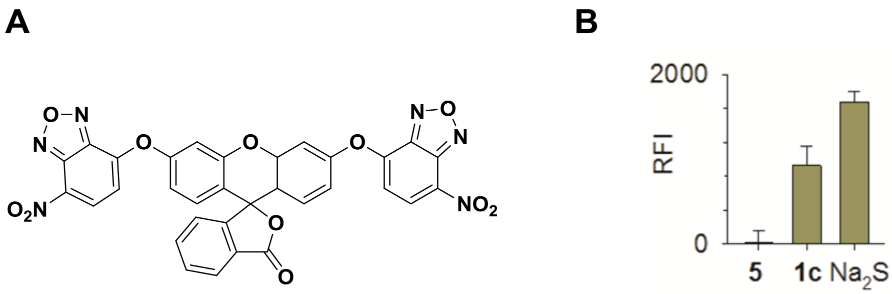

Figure S1. A) Structure of NBD-fluorescein. B) Hydrogen sulfide generated during incubation of **1c** with NTR in HEPES buffer pH 7.4 was estimated using NBD-Fluorescein.

Time course of H_2S release from 1c: A stock solution of **1c**, **4a,** NADH, and *E. coli* nitroreductase (NTR) were prepared as mentioned above. A reaction mixture was prepared by adding **1c** (4 µL, 2.5 mM), NADH (10 µL, 1 mM) and NTR (4 µL) in HEPES buffer (162 µL) pH 7.4. The resulting mixture was incubated at 37 °C. At different time interval (5, 15, 30 and 45 min) 20 µL (1 mM) **4a** was added to the above mixture and incubated under dark conditions for 10 min at 37 °C. The fluorescence (excitation 444 nm; emission 520 nm) from reaction mixture was measured using microtiter plate reader. A calibration curve was used to quantify hydrogen sulfide produced during incubation of **1c** with NTR. In the control experiment **1c** (4 µL, 2.5 mM) was incubated with NADH (10 µL, 10 mM) in HEPES buffer (166 µL) pH 7.4 and at different time interval **4a** (20µL, 1 mM) was added. The fluorescence from reaction mixture was measured. Data presented are an average of three independent experiments.

Chemoreduction of 1e with Zn and ammonium formate: Compound **1e** (10 µL, 10 mM) was dissolved in 980 µL of a 1:1 solution of methanol and phosphate buffer (10 mM, pH 7.4). To this mixture, ammonium formate (10 µL, 100 mM) and zinc dust (1 mg) were added and the reaction mixture was incubated at 37 ºC for 30 min. The reaction mixture was filtered (0.22 µm) and injected (50 µL) in a high-performance liquid chromatograph (HPLC) attached with a diode-array detector (the detection wavelength was 250 nm) and a Zorbax SB C-18 reversed-phase column (250 mm × 4.6 mm, 5 µm). A mobile phase of water/acetonitrile (25: 75) was used with a flow rate of 1 mL/min for 20 min. The authentic compound **2e** was injected to identify the peak position of the reaction product. Two independent experiments were conducted, each carried out in duplicate and representative data is presented below.

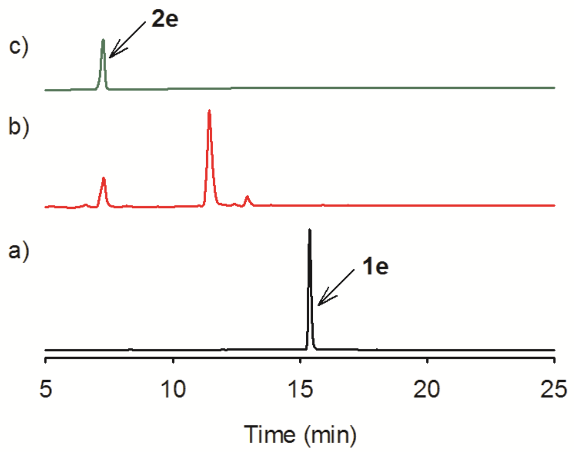

Figure S2. Representative HPLC traces of chemoreduction of **1e** using Zn and ammonium formate in MeOH: pH 7.4 phosphate buffer (1 mL, 1:1 v/v) after 30 min: a) Authentic **1e** (100 µM) in acetonitrile; (Note: The peak at RT 12 min is likely the partially reduced compound, but we were unable to characterize it) b) 100 µM reaction mixture after 30 min; c) Authentic acetophenone, **2e** (100 µM) in acetonitrile.

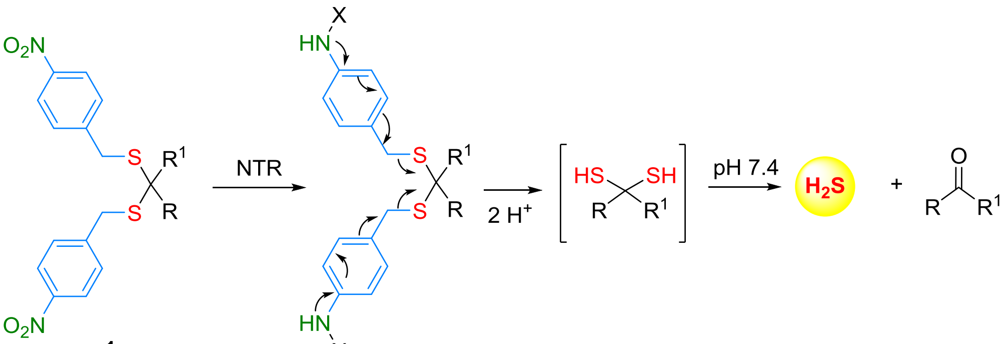

Scheme S2. Proposed mechanism for H_2S generation from bis(4-nitrobenzyl)sulfanes

Intracellular detection of H_2S from 1c using HPLC: *Escherichia Coli* (ATCC 25922) was cultured in 5 mL of tryptone soya broth (TSB) medium at 37 ºC for 16 h. The cultured bacteria were centrifuged to aspirate out the medium and resuspended to an O.D of 1.0 with fresh TSB medium. This bacterial solution was incubated with 50 μM of **1c** and 10 μM of azo-BODIPY (**4a**) for 60 min in dark by covering the falcon tube in an aluminium foil. The suspension was centrifuged to aspirate out any excess of the compound and/or azo-BODIPY in the medium. The bacterial pellet was washed with HEPES buffer pH 7.4 (1 mL × 3) and centrifuged. The collected bacterial pellet was re-suspended with acetonitrile and the cells were lysed by vortexing for 1 min. The cell lysate was then removed by centrifugation and the supernatant acetonitrile (50 μL) was injected in an Agilent high performance liquid chromatography (HPLC) attached with a fluorescence detector (excitation at 444 nm; emission at 520 nm). The HPLC method used was as described previously.

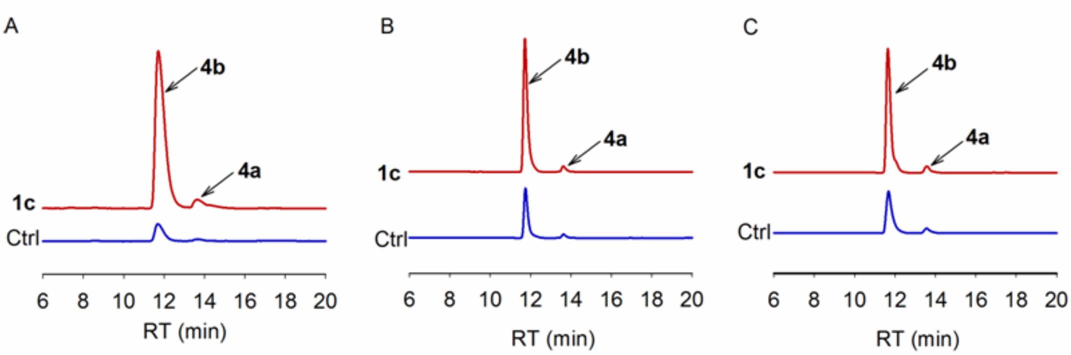

Figure S3. H_2S generation during incubation of bacteria with **1c** was determined by HPLC analysis of the cell lysate; (a) *E.coli*, (b) *B. subtilis*, (c) *M. smegmatis*

FACS analysis: *E. coli* cells were grown in luria broth (LB) until mid-exponential phase (OD_{600} ~ 0.6) and just before assay cells were diluted to an OD_{600} of 0.2. The *E. coli* cells were washed with 1xPBS and suspended in LB. Cells were pre-treated with **1c** (50 μM) for 20 min followed by addition of **4a** (10 μM) for 5 min. The cells were also treated with **5** (50 μM) as a negative control and sodium sulphide (50 μM) as a positive control. The H_2S levels were monitored by measuring mean fluorescence intensity of 30,000 *E coli* cells at an excitation

wavelength of 405 nm and emission wavelength of 520 nm using a BD FACS verse flow cytometer (BD Biosciences). Data was analyzed using the FACSuite software.

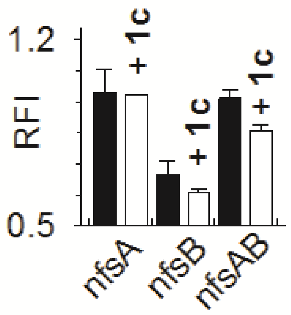

Figure S4. H_2S generation using H_2S-sensitive dye **4a** in *E. coli nfsA*, *nfsB* and *nfsAB* mutants that lacked nitroreductase genes. RFI is normalized relative fluorescence intensity

H_2S generation in macrophages: The human monocytic cell line THP-1 and Raw 264.7 cells were maintained in an atmosphere containing 5% CO_2 at 37 °C in the culture medium recommended by ATCC. The THP-1 and Raw 264.7 cells were harvested and washed in 1x phosphate buffer saline (1xPBS) and suspended in 1x PBS containing 3% fetal bovine serum. Cells were pre-treated with **1c** (50 µM) for 30 min followed by addition of **4a** (10 µM) for 5 min. The sodium sulfide (50 µM) was used as positive control. The H_2S levels were monitored by measuring mean fluorescence intensity of 30,000 THP-1 cells at an excitation wavelength of 405 nm and emission wavelength of 520 nm using a BD FACS verse flow cytometer (BD Biosciences). Data was analyzed using the FACSuite software.

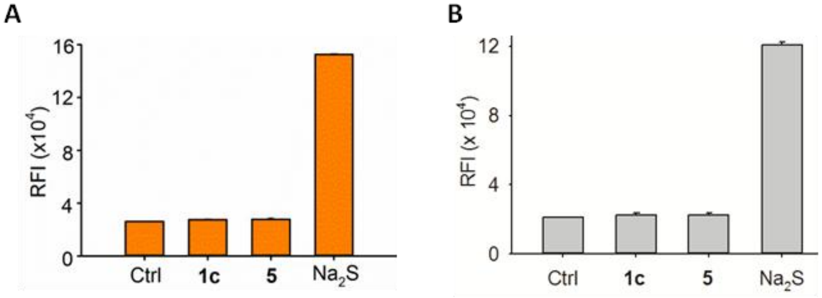

Figure S5. H_2S generation in: (a) RAW 264.7 cells and (b) U937 cells.

Oxidative stress measurement using roGFP2 redox sensor To examine the effect of H_2O_2 or antibiotics-induced oxidative stress exposure and protective role of H_2S, we used variable concentrations of Ampicillin, Amikacin and Ciprofloxacin for specific time points. roGFP2 expressing *E.coli* cells were firstly grown aerobically till mid-exponential phase (OD_{600} of 0.6) and diluted to OD_{600} of 0.2 followed by pre-treatment with **1c** for 20 min. Specific concentration of antibiotics were used and biosensor response (405/488 ratio at a fixed emission of 520 nm) was measured. Increase in 405/488 ratio indicated that antibiotic exposure leads towards the oxidative stress induction.

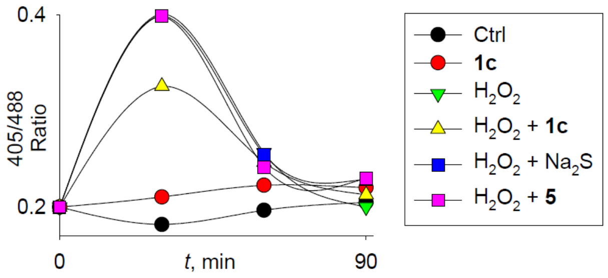

Figure S6. Reduction-oxidation sensitive GFP (roGFP2) was used to measure dynamic changes in cytoplasmic redox potential of *E. coli* upon exposure to: H_2O_2, 1 mM; **1c**, 100 μM; Na_2S, 100 μM and **5**, 100 μM.

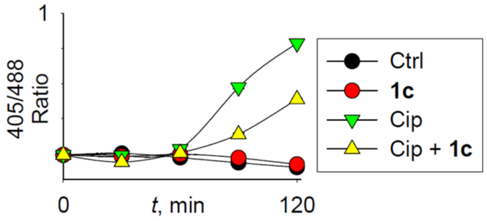

Figure S7. Dynamic changes in cytoplasmic redox potential of *E. coli* upon exposure to Cip 5 μg/mL; **1c**, 100 μM.

Time-kill assay. Cells were grown aerobically till mid-exponential phase (OD_{600} of 0.6) and diluted to OD_{600} of 0.2 in LB-broth and pre-exposed to **1c** (100 μM) was given for 20 min. Antibiotics and H_2O_2 treatments were given for certain time points with their selective concentrations. For survival analysis, serial dilutions of cultures were plated at each time points on LB- Agar plates and plates were maintained at 37°C. CFUs were counted carefully and the results were calculated as bacterial survival percentage against their respective untreated control.

qRT-PCR analysis: *E. coli* cells were grown till mid-exponential phase (OD_{600} of 0.6) and diluted to OD_{600} of 0.2, followed by pre-treatment with **1c** for 20 min. Antibiotic treatment was given for 1h and total RNA was isolated using Trizol method. First-strand cDNA synthesis was performed using 200 ng of the total RNA with iScript Select cDNA Synthesis Kit (Bio-Rad) using random oligonucleotide primers. PCR was performed using gene specific primers (Table S2). Gene expression was analyzed with real-time PCR using iQTM SYBR GreenSupermix (Bio-Rad) and a CFX96 RT-PCR system (Bio-Rad). Data analysis was performed with the CFX ManagerTM software (Bio-Rad).

Table S1. Sequences of primers used in this study

Gene Name	Primer Sequence (for qRT-PCR)
cyoA	F: 5' CGAGAAGCCCATTACCATCG 3'<br>R: 5' CGGAGTTGGAGGTCACTTTG 3'
cydB	F: 5'CCGGTAACTTCTTCCAGTTG3'<br>R: 5' GGTACGCATTTGCAGATAGG3'
appY	F: 5'GAAGGGAGTTACCAGTATGC 3'<br>R: 5' TGCCAGGCAGCTCATTATTC 3'
16s rRNA	F: 5' CAGGTGTAGCGGTGAAATGC 3'<br>R: 5' ACATCGTTTACGGCGTGGAC3'

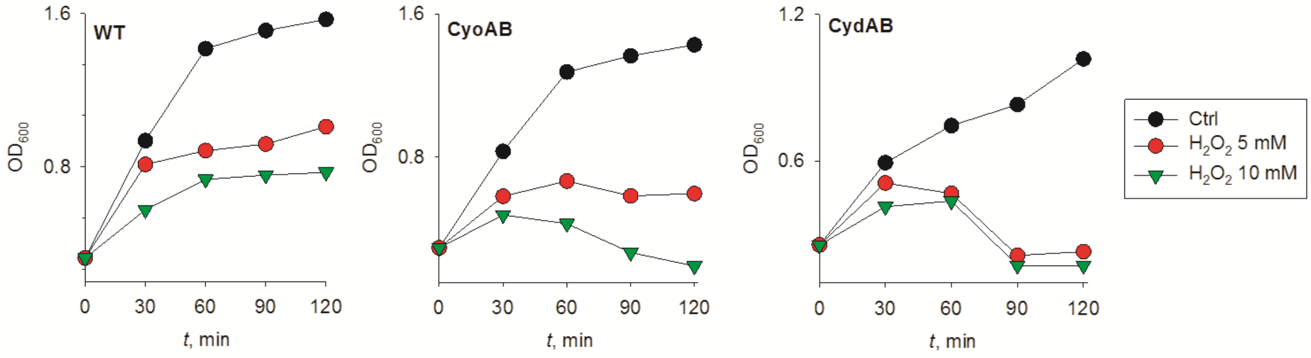

Figure S8. Growth curves for *E. coli* strains in the presence of H_2O_2. This data supports heightened sensitivity of *cydAB* mutant towards H_2O_2.

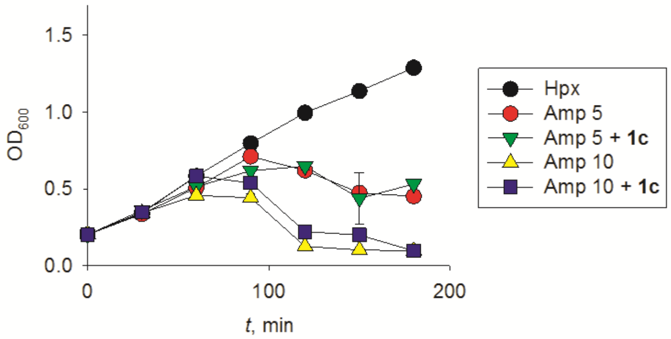

Figure S9. Growth curves for *E. coli* strains in the presence of Amp or Amp + **1c**. Amp 5 indicates 5 μg/mL while Amp 10 indicates 10 μg/mL. This data suggests that H_2S is ineffective in protecting from Amp-induced stress in the Hpx mutant.

Table S2. Strains used for the experimental studies

Isolate	Ampicillin (50 µg/ml)	Streptomycin (100 µg/ml)	Tetracycline (60 µg/ml)	Nalidixic acid (100 µg/ml)	Kanamycin (50 µg/ml)	TEM PCR	*bla*$_{TEM}$ GenBank accession	CTX PCR	*bla*$_{CTX-M-15}$ GenBank accession
P12	+	+	+	+	+	Negative	NA	Positive	KY568704
P14	+	+	+	+	+	Positive	NA	Positive	NA
P20	+	+	+	+	+	Negative	NA	Positive	KY568702
P21	+	+	+	+	-	Positive	NA	Positive	NA
P28a	+	+	+	+	+	Negative	NA	Positive	KY568703
P45	+	+	+	+	+	Negative	NA	Positive	KT956439
Q10	+	+	+	+	+	Positive	NA	Positive	NA
Q40	+	+	+	+	+	Positive	NA	Positive	NA

NA - not available, Presence of TEM-β-lactamase or CTX-M-β-lactamase was confirmed by PCR.

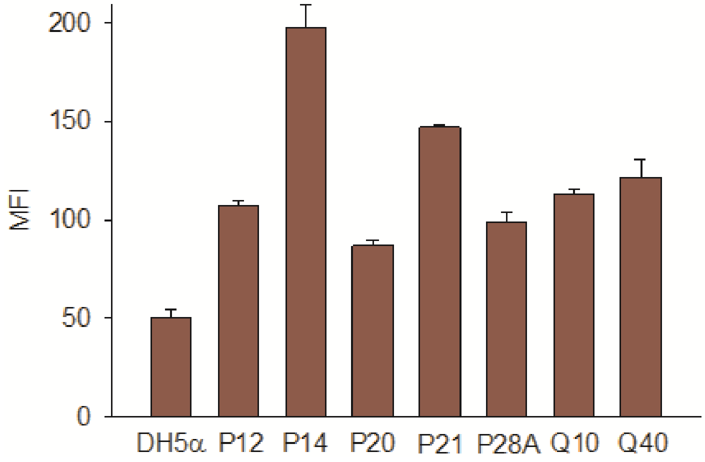

Figure S10. H_2S levels in multi-drug resistant strains of uropathogenic *E. coli*. DH5α is wild-type strain while the others are patient-derived urinary tract infection causing *E. coli* strains. Statistical analysis of relative H_2S levels between DH5α and each of the individual multi-drug resistant strains showed p-values ranging from 0.0008-0.0095.

References:

1 M. J. Robins, Y. Peng, V. L. Damaraju, D. Mowles, G. Barron, T. Tackaberry, J. D. Young and C. E. Cass, *J. Med.Chem.*, 2010, **53**, 6040.

2 T. Saha, D. Kand and P. Talukdar, *Org. Biomol. Chem.*, 2013, **11**, 8166.

3 K. Sharma, K. Sengupta and H. Chakrapani, *Bioorg. Med. Chem. Lett.*, 2013, **23**, 5964.

NMR Spectra

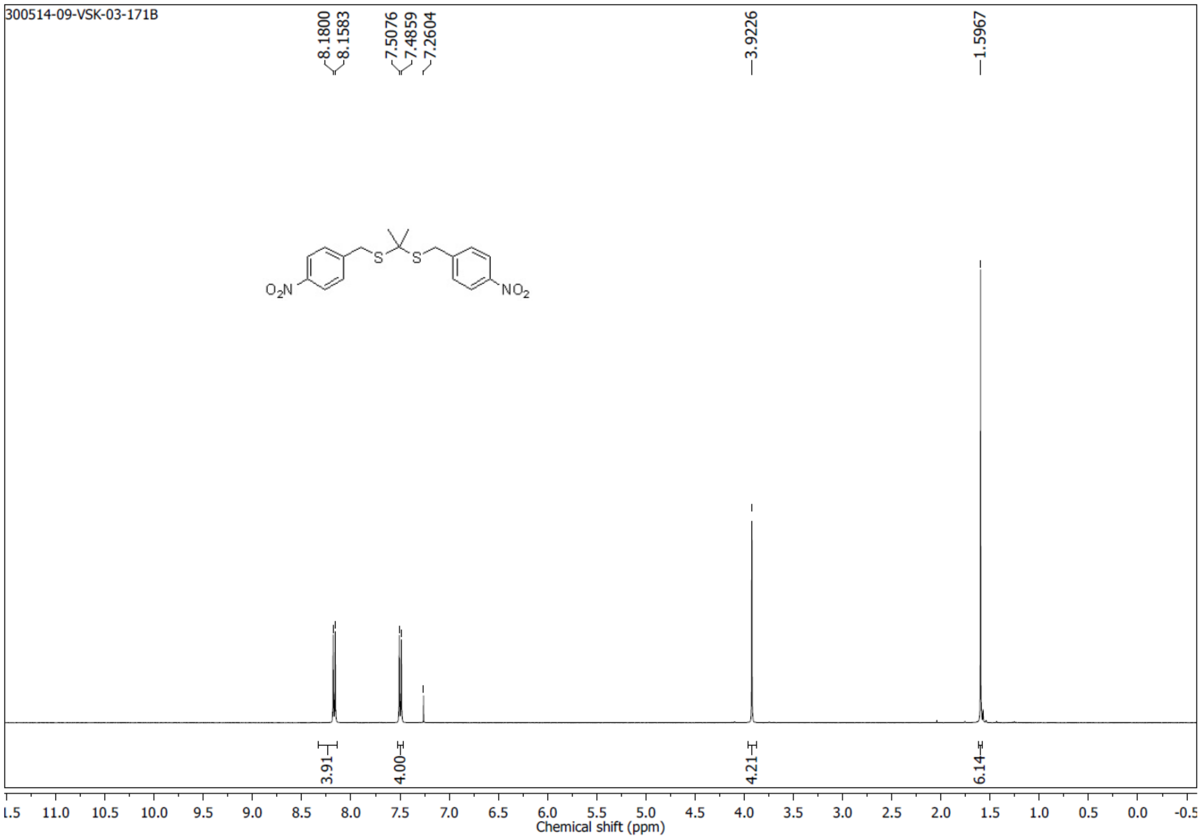

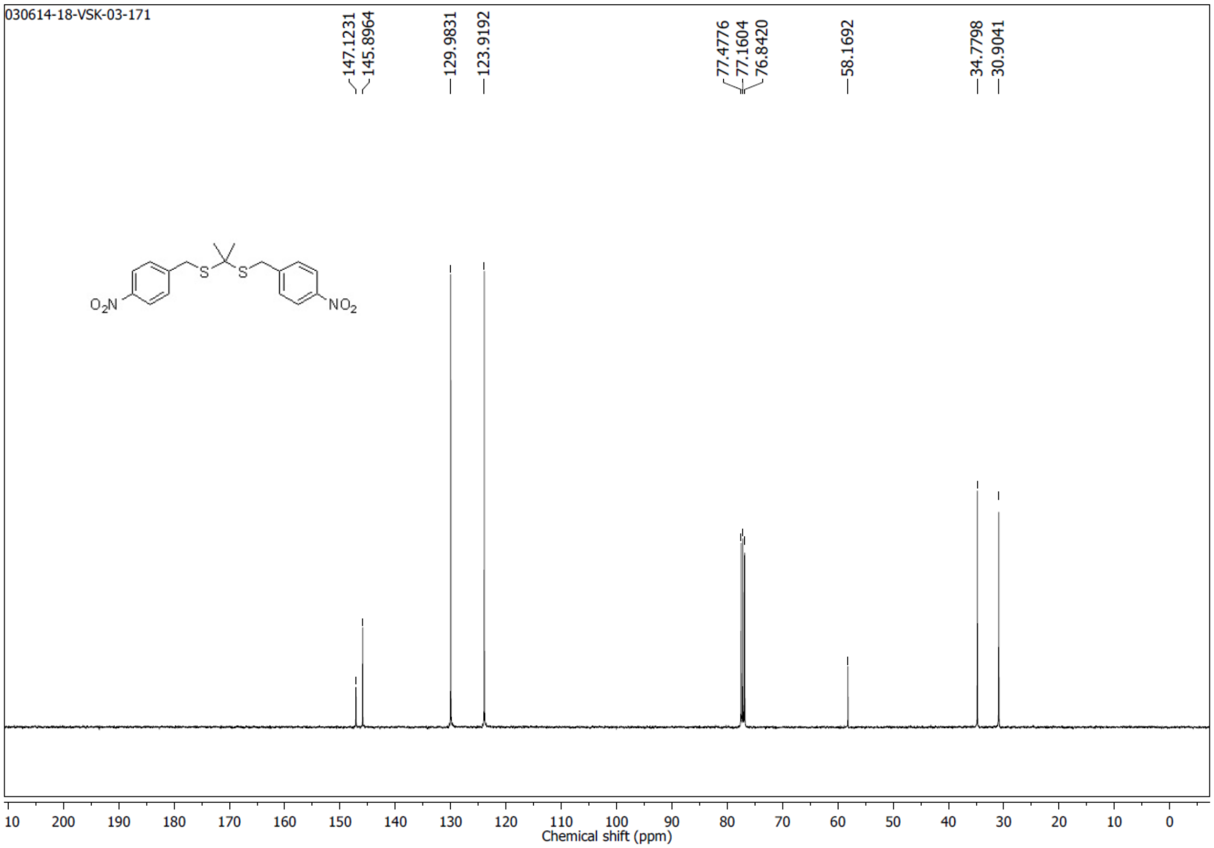

^{1}H and ^{13}C NMR spectra of **1a**

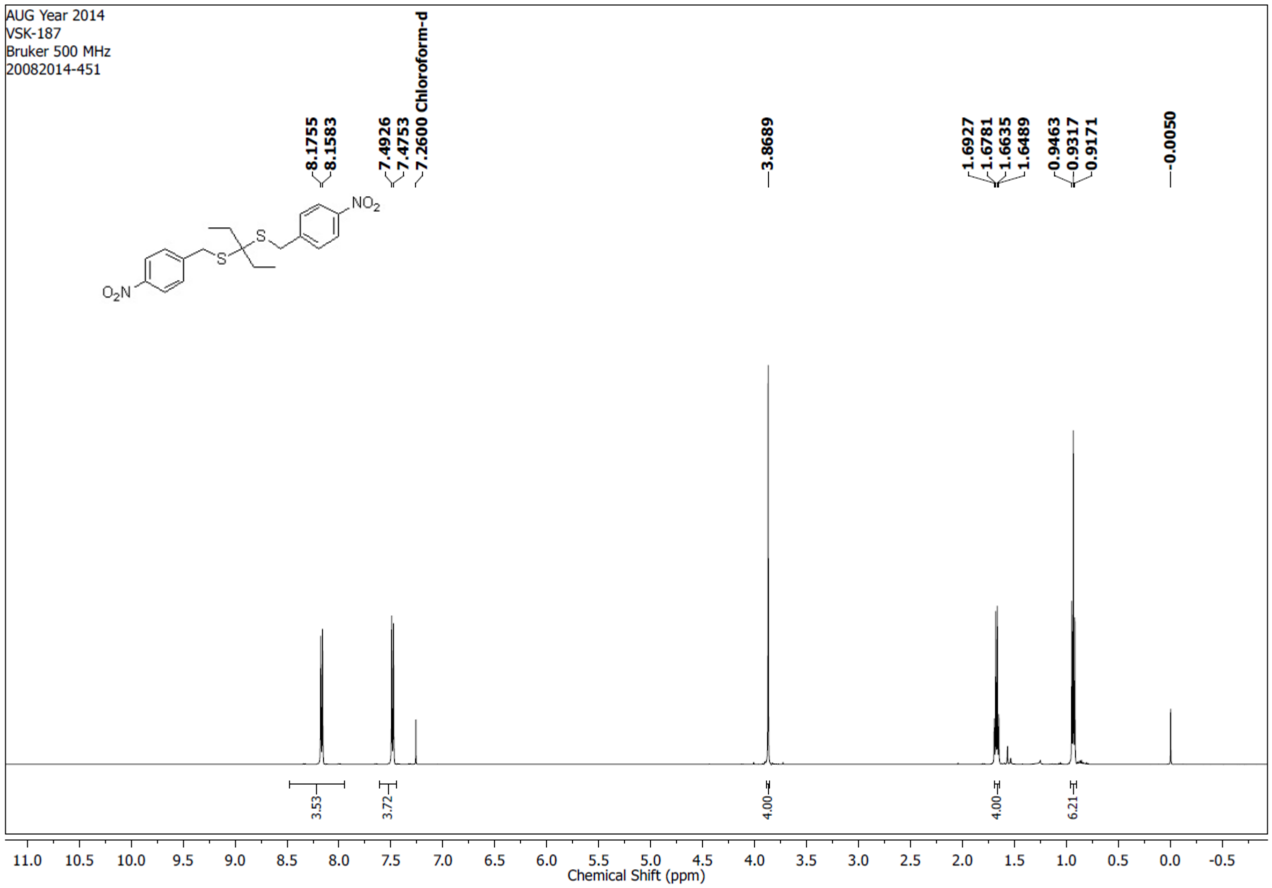

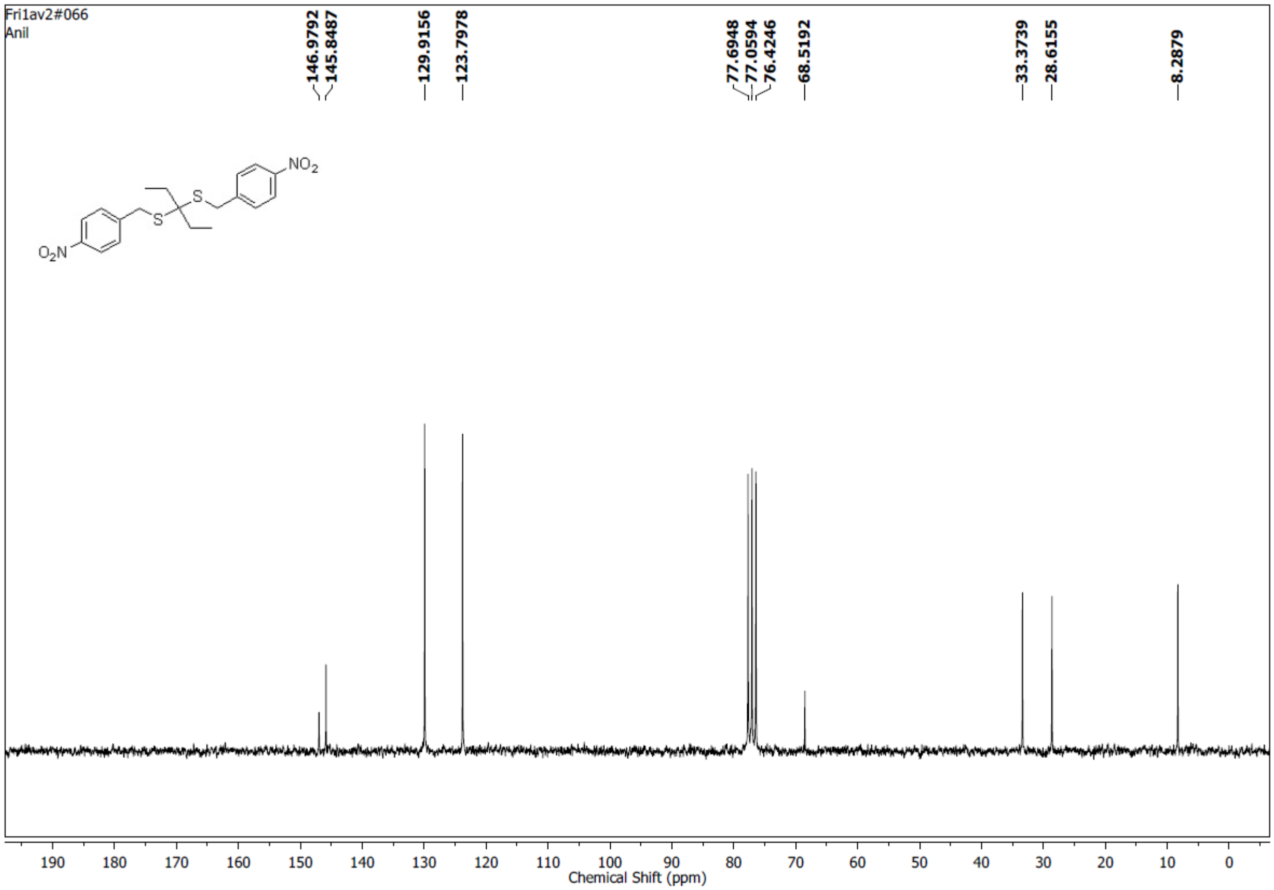

^{1}H and ^{13}C NMR spectra of **1b**

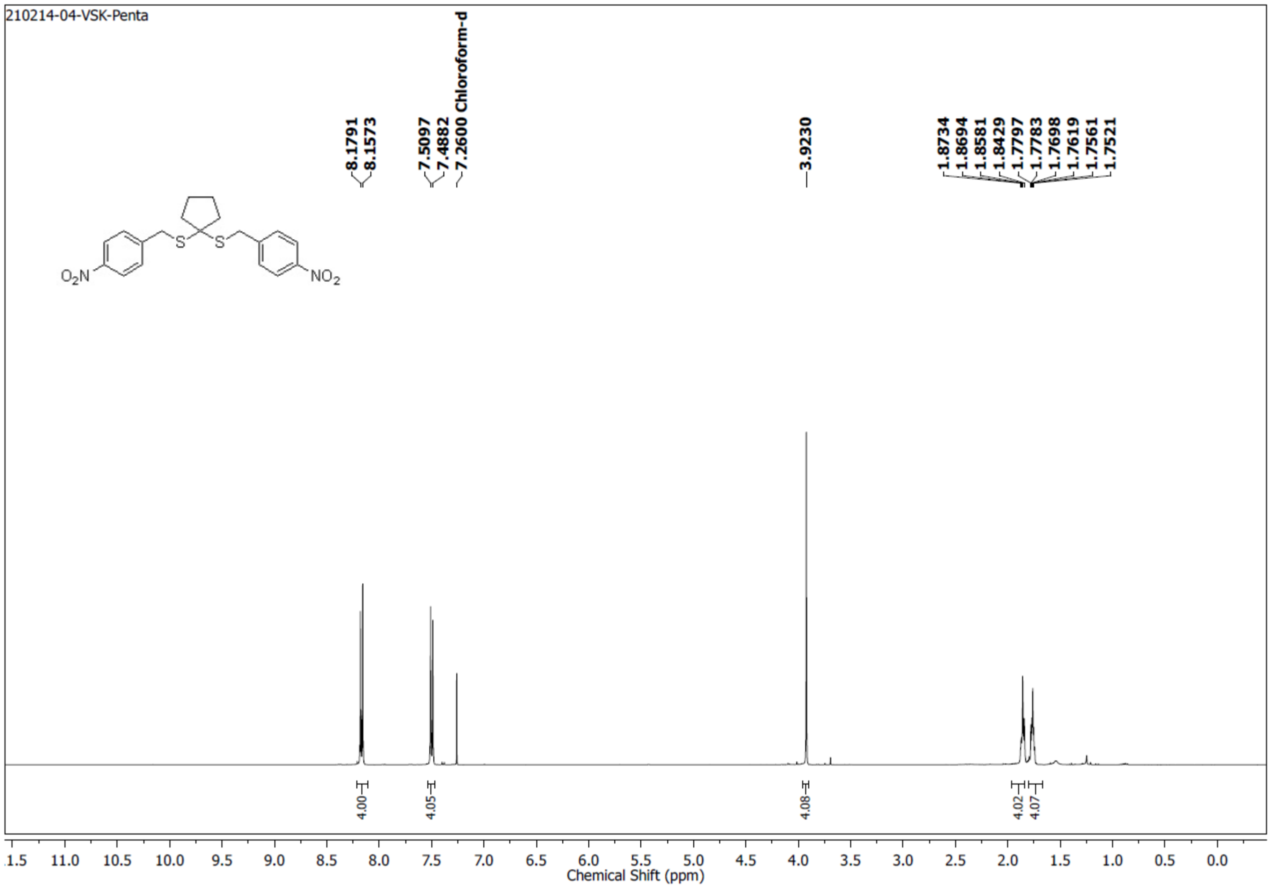

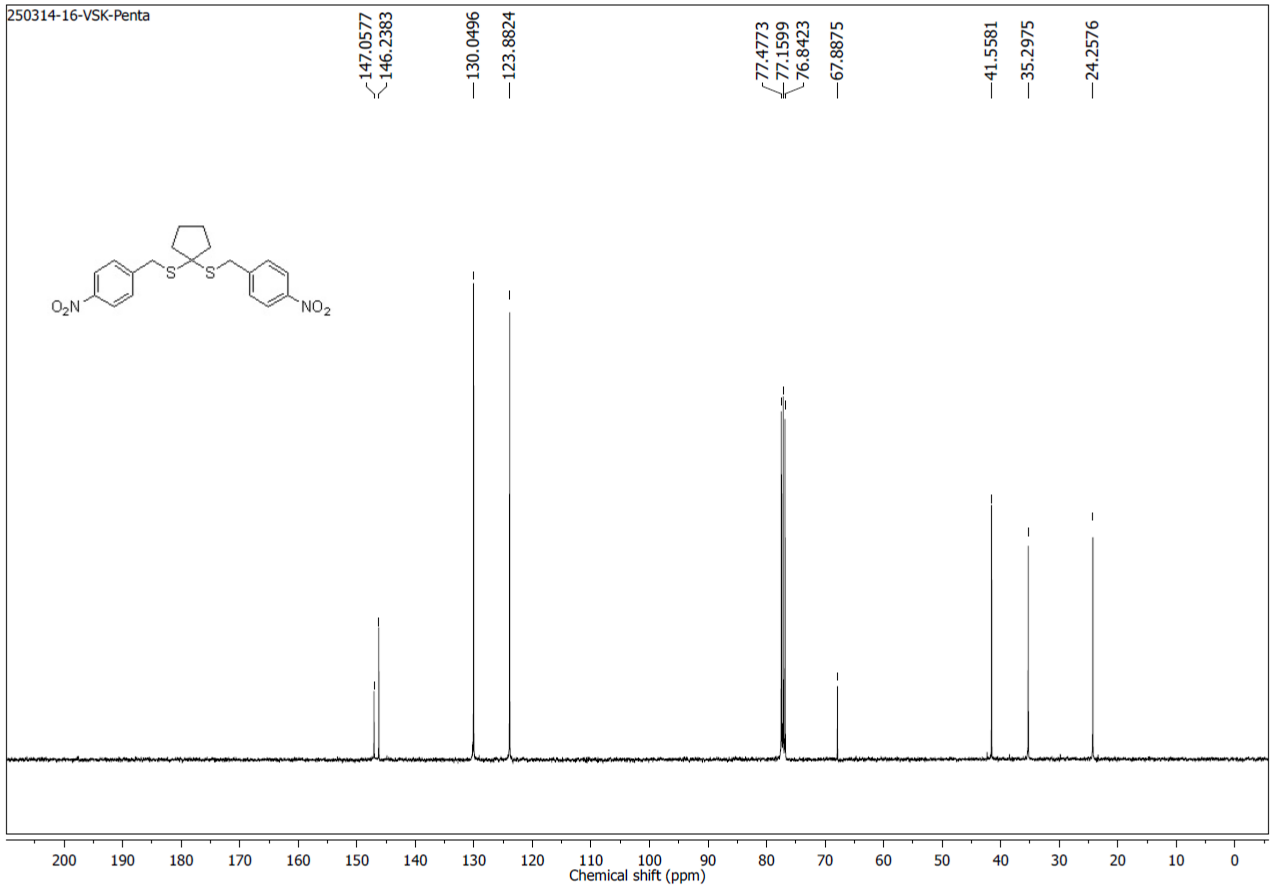

^{1}H and ^{13}C NMR spectra of **1c**

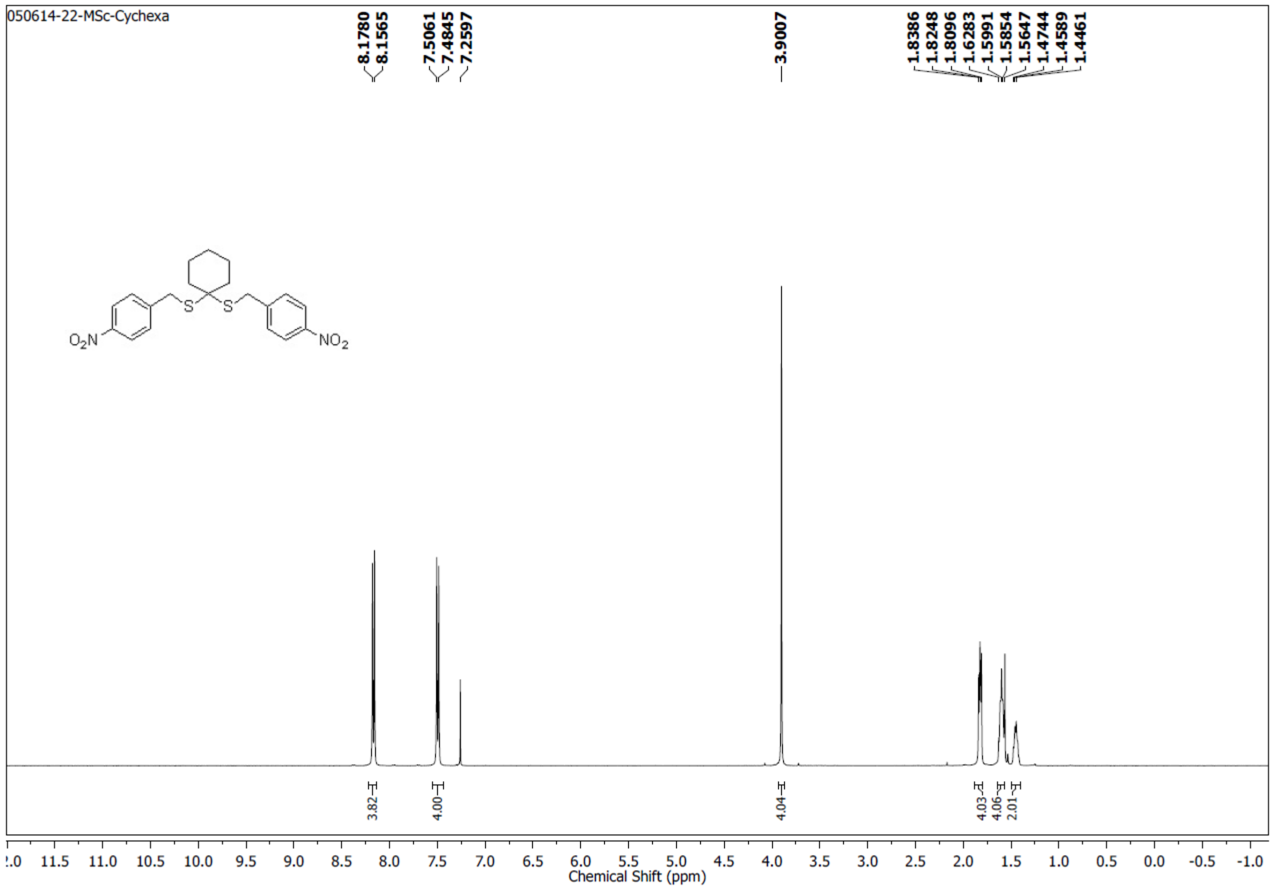

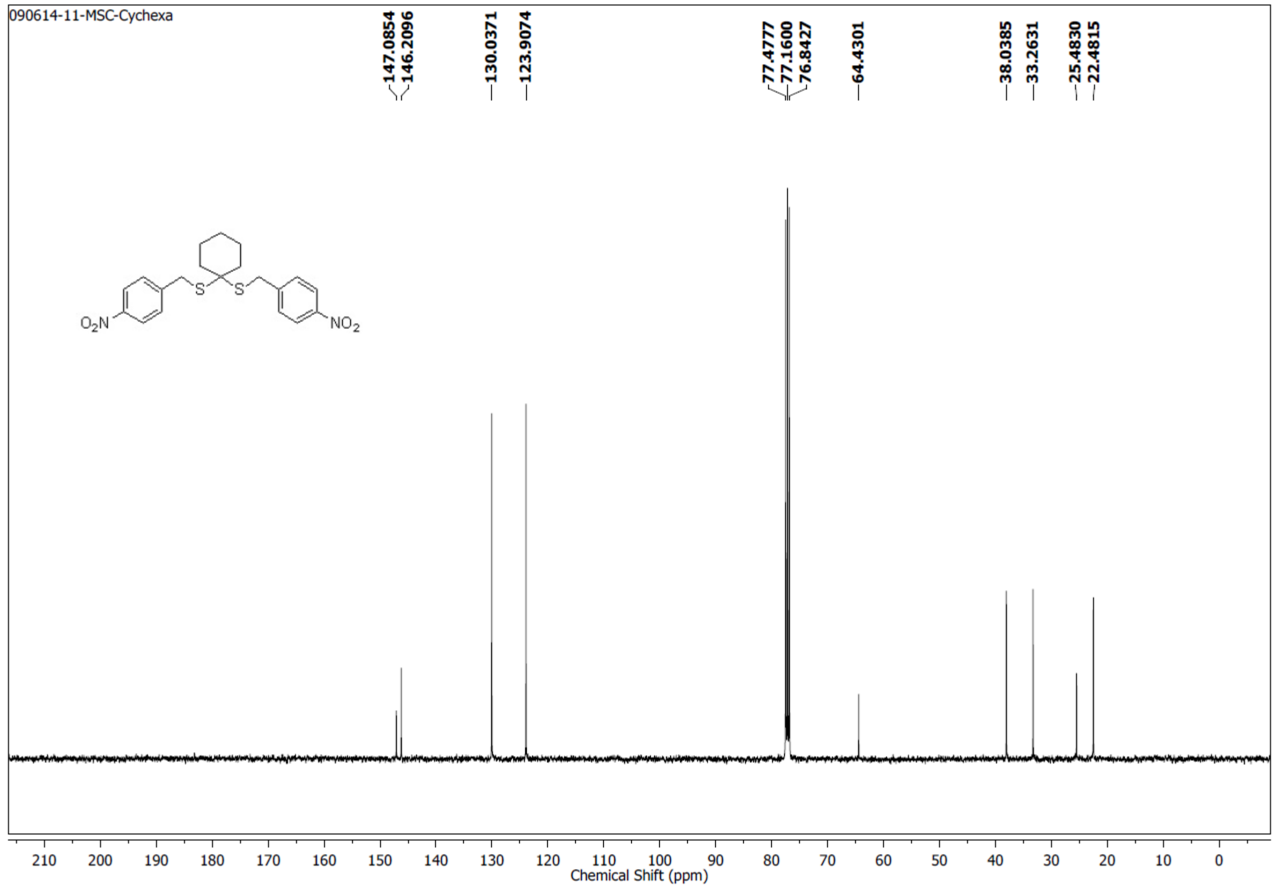

^{1}H and ^{13}C NMR spectra of **1d**

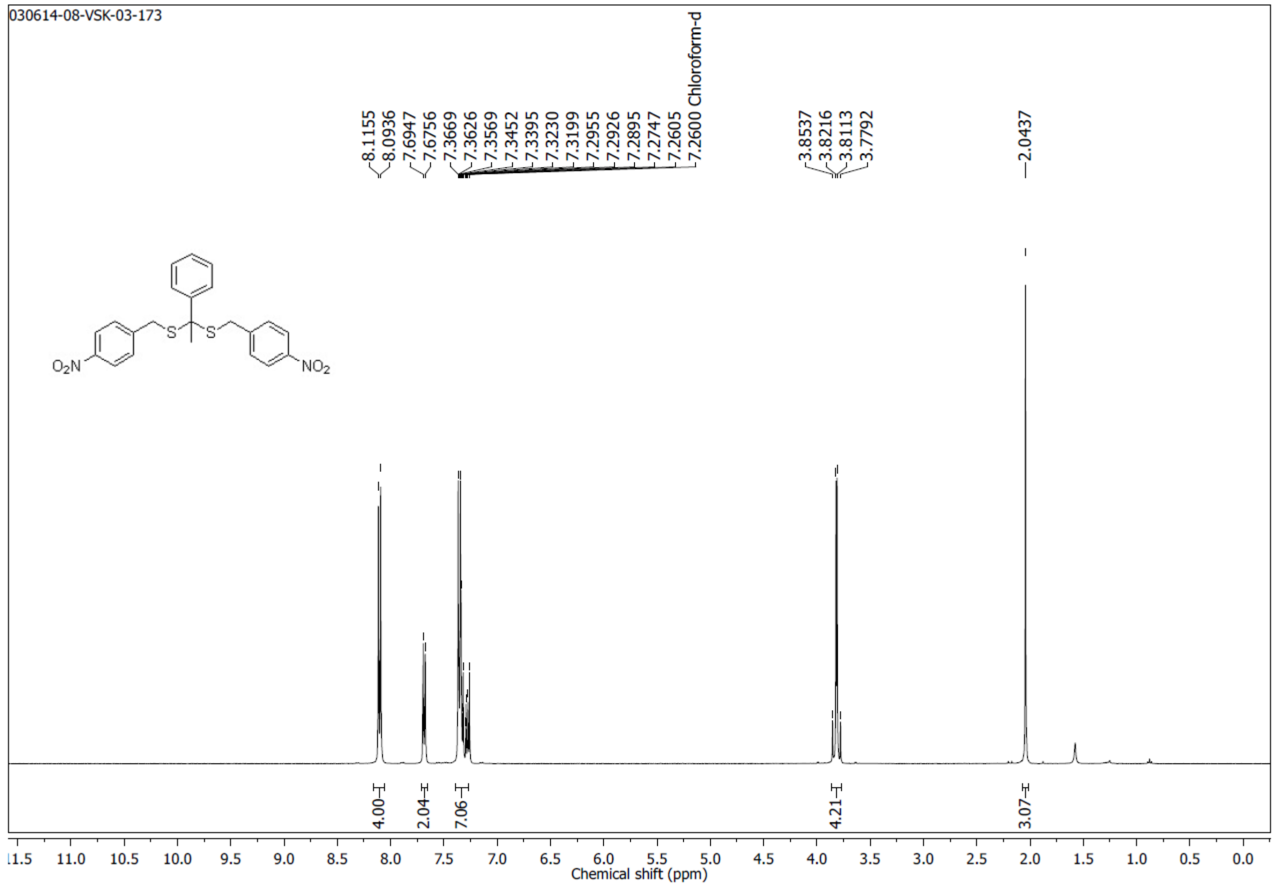

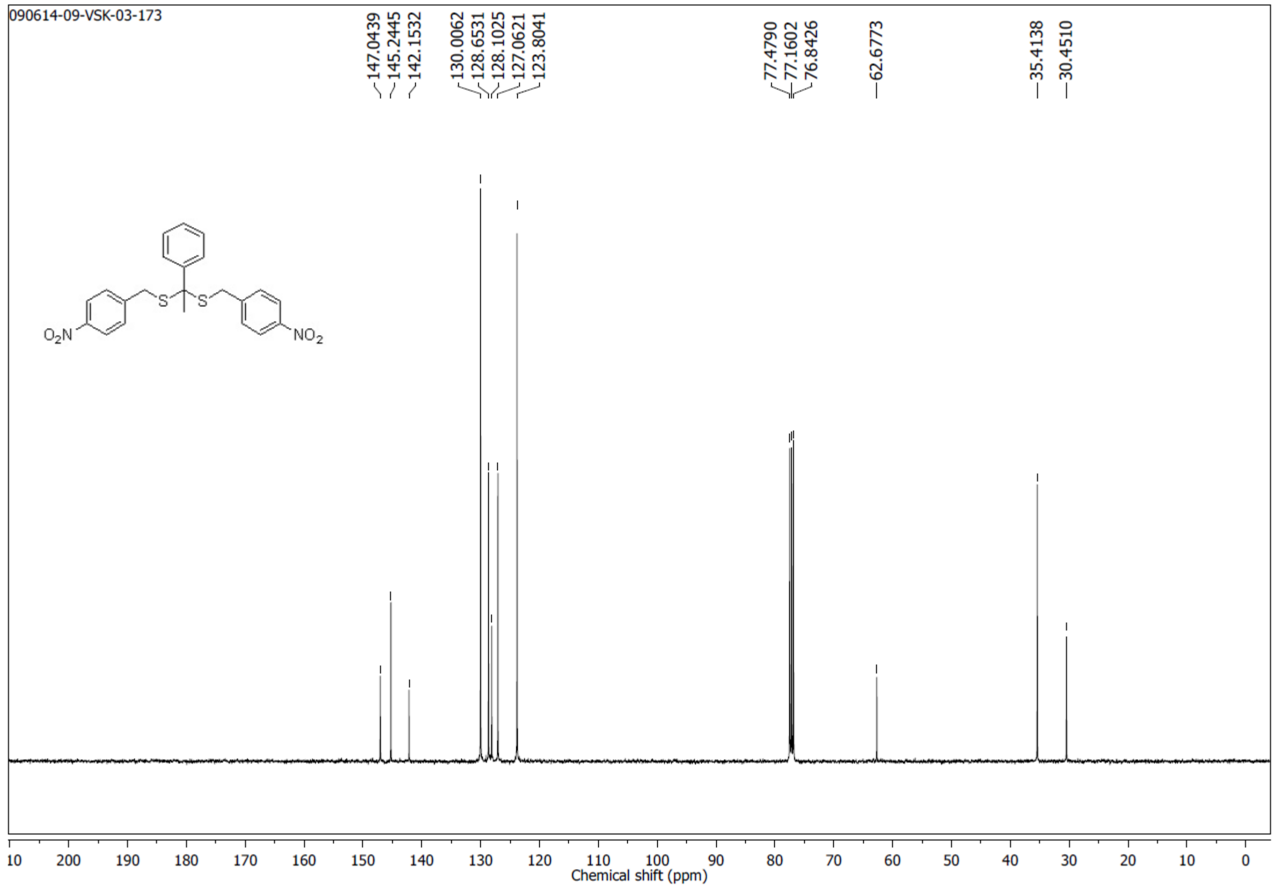

^{1}H and ^{13}C NMR spectra of **1e**

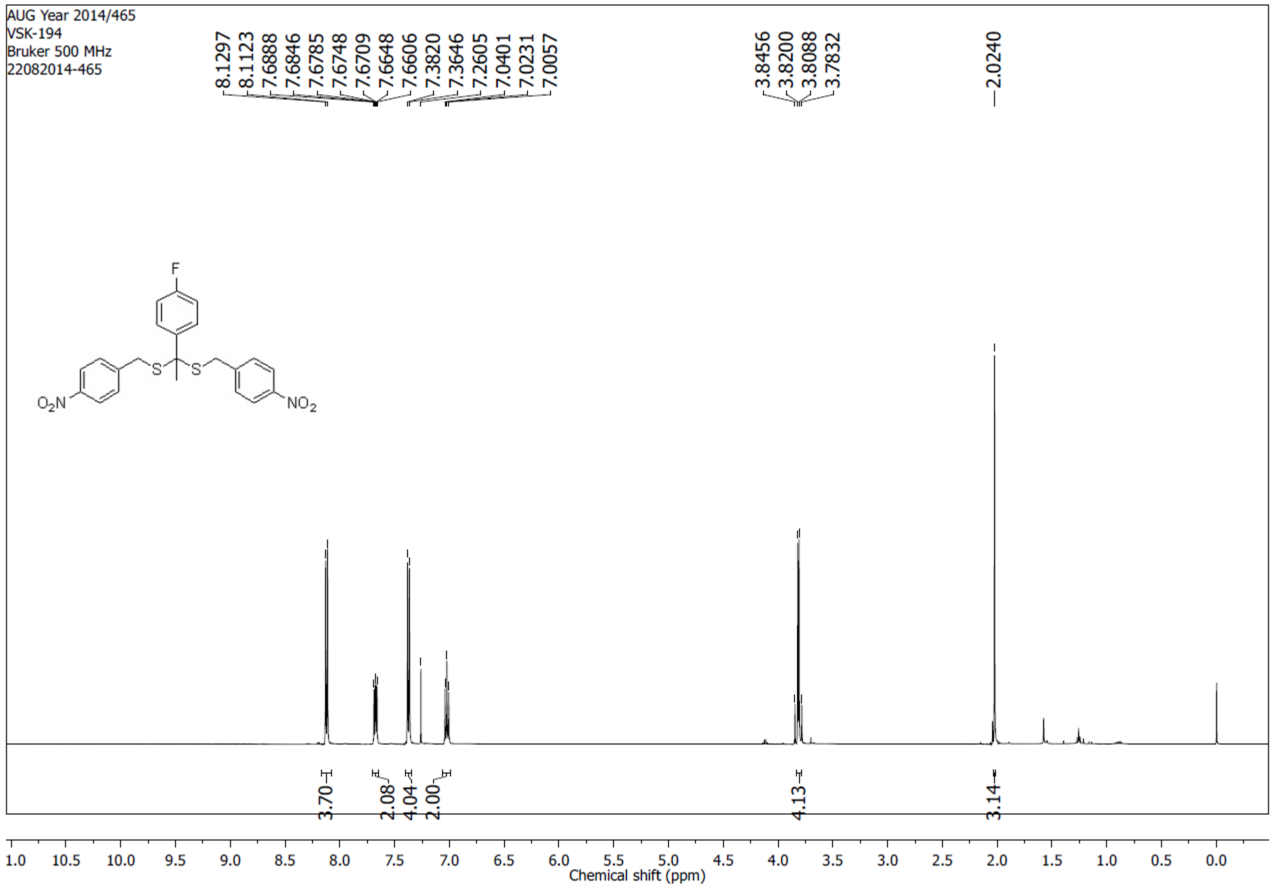

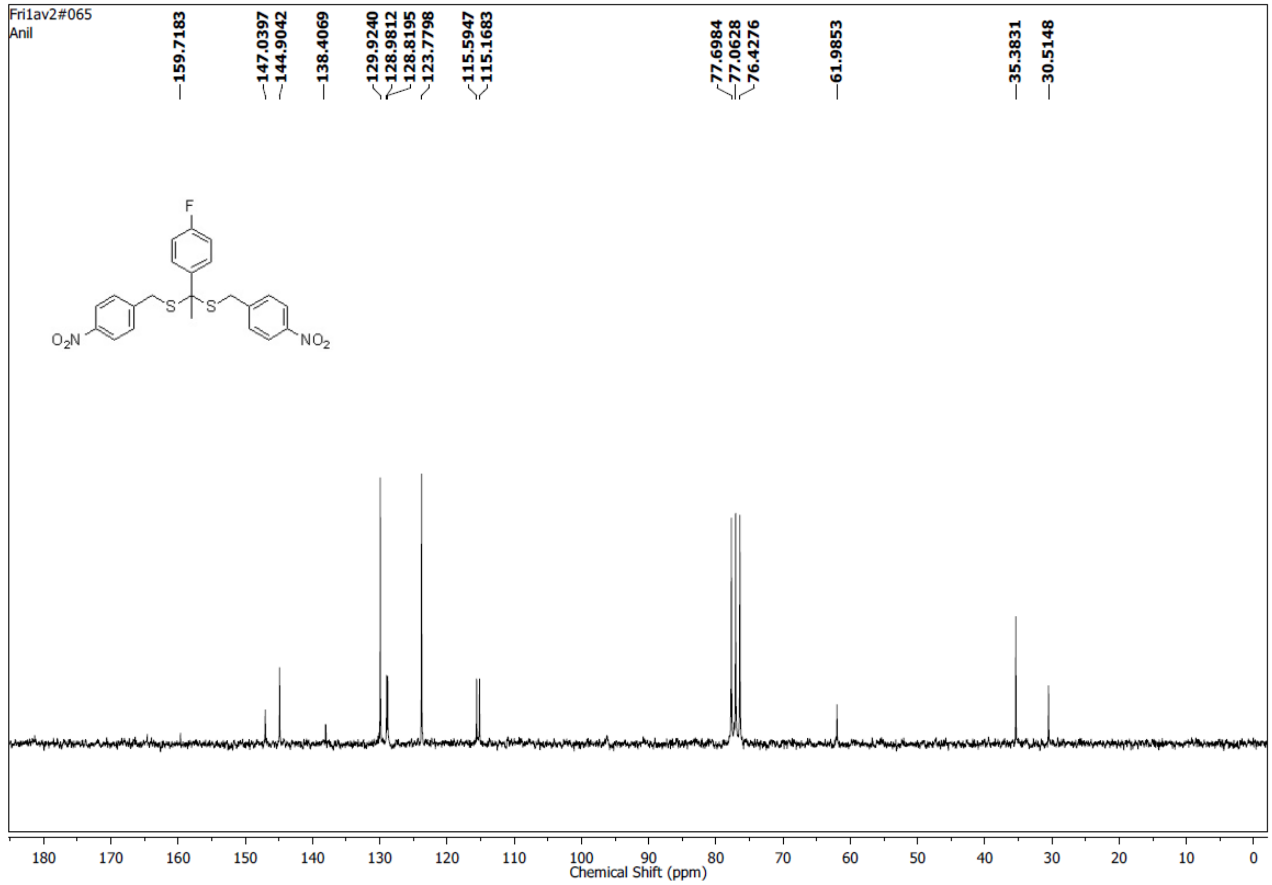

^{1}H and ^{13}C NMR spectra of **1f**

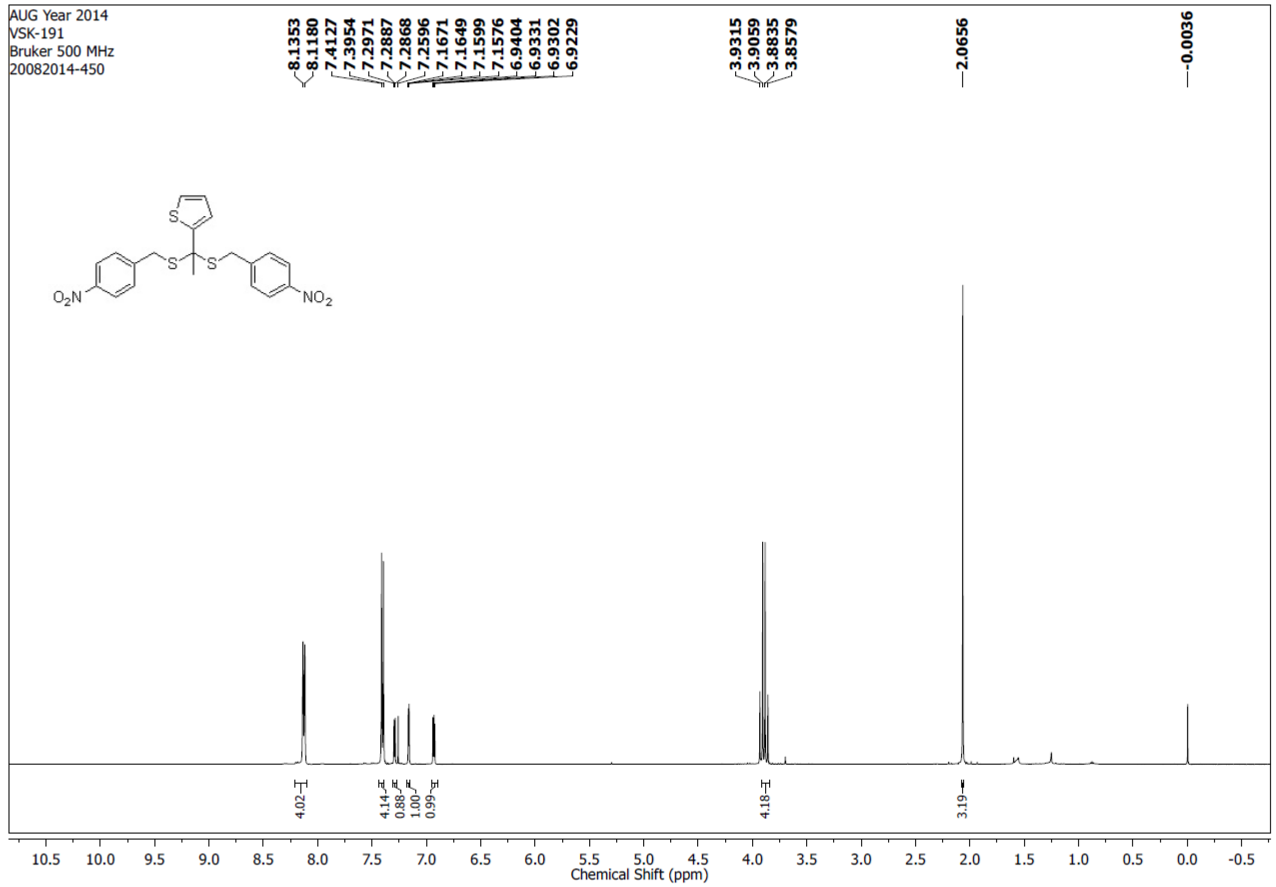

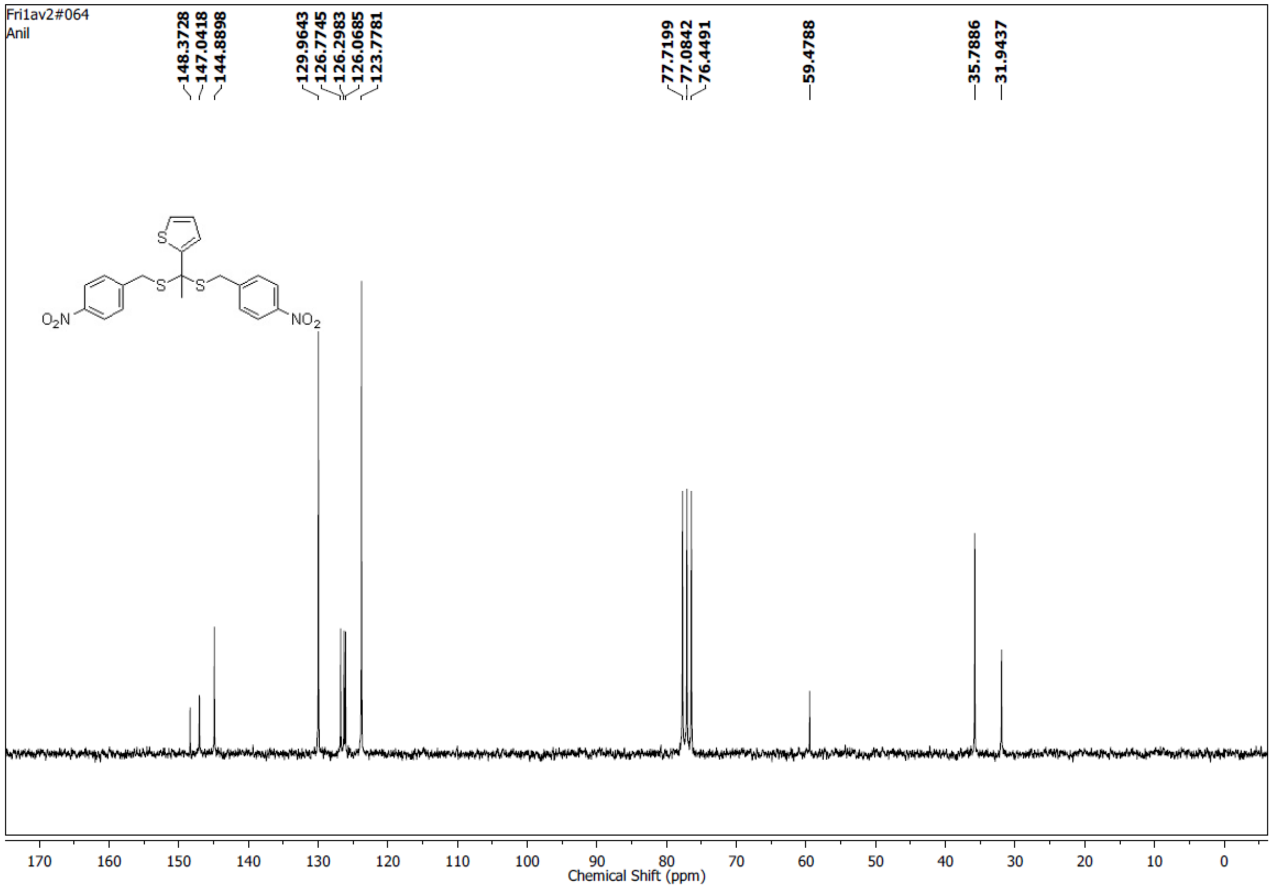

^{1}H and ^{13}C NMR spectra of **1g**

HPLC traces of compounds

1a

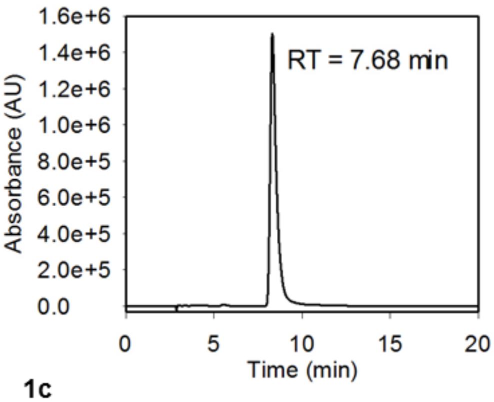

1b

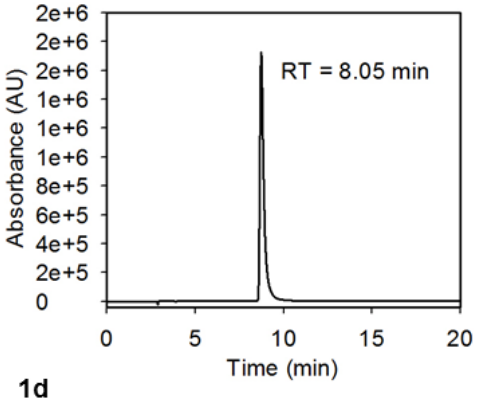

1c

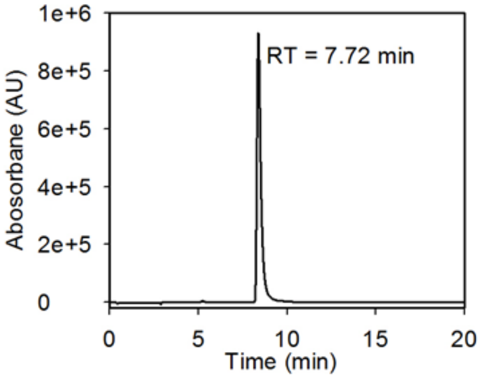

1d

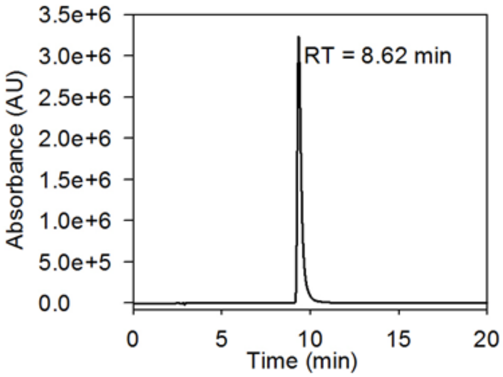

1e

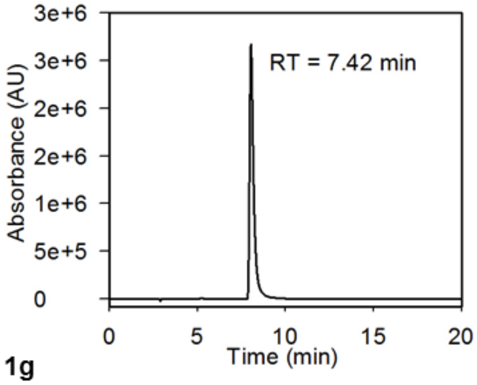

1f

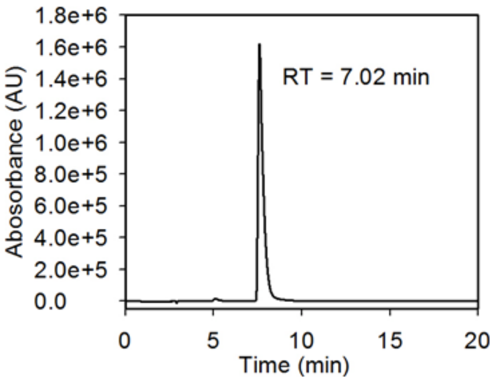

1g

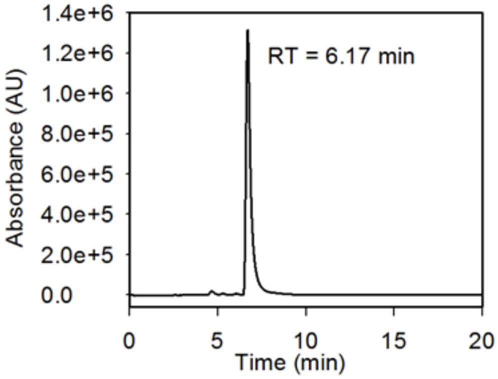

Supplement: Supplementary file 1 [file SC-008-C7SC00873B-s001.pdf]
